# Cell Painting in activated cells illuminates phenotypic dark space and drug mechanisms of action

**DOI:** 10.1038/s41467-026-76252-6

**Published:** 2026-08-14

**Authors:** Matylda A. Zietek, Akshar Lohith, Derfel Terciano, Beverley M. Rabbitts, Aswad Khadilkar, John B. MacMillan, R. Scott Lokey

**Affiliations:** 1 Department of Chemistry and Biochemistry, University of California, Santa Cruz, Santa Cruz, CA USA; 2 Department of Biomolecular Engineering, University of California, Santa Cruz, Santa Cruz, CA USA; 3 Chemical Screening Center, University of California, Santa Cruz, Santa Cruz, CA USA

**Keywords:** High-throughput screening, Phenotypic screening, Target identification

## Abstract

As drug and natural product libraries expand, assays for assessing mechanisms of action (MoA) are increasingly critical. Performing cytological profiling using the Cell Painting (CP) assay enables image-based profiling of cellular states upon treatment, yet many bioactive compounds remain uncharacterized due to undetectable cellular effects under standard conditions. To address this, we combine drug dosing with cell activation using the protein kinase C (PKC) agonist phorbol myristate acetate (PMA). Profiling A549 lung cancer cells treated with 8,387 compounds at two concentrations (1 and 10 µM) in both resting and PMA-activated states enables detection of phenotypic effects for up to 40% of all screened compounds, effectively illuminating more phenotypic ‘dark space.’ The combined cellular CP fingerprints from PMA-activated and resting cells reveal phenotypes for 565 compounds that are inactive under the resting condition alone, supporting its use in MoA studies. We introduce quality control measures for CP screens and demonstrate that integrating phenotypic signatures supports MoA discovery. Notably, 2-methoxycinnamaldehyde clusters with glucocorticoid receptor modulators and induces nuclear translocation, emphasizing the utility of this approach in identifying drug mechanisms and, therefore, aiding in improving therapeutic strategies.

## Abstract (web summary)

In this work, researchers show that activating cells before imaging reveals drug effects undetected under standard conditions, identifying unreported phenotypes for hundreds of compounds and identifying a natural product as a glucocorticoid receptor modulator.

## Introduction

A living cell contains myriad complex processes and structures operating concurrently. At any moment, multiple activities coordinate across organelles in an intricately dynamic system. The cell’s interaction landscape depends on conditions such as developmental stage, nutrient availability, and signaling status. Encountering a signaling molecule or stress can induce the cell to undergo physiological and morphological changes—for example, transitioning from a resting to an activated state. This dynamic landscape can be described using various information-rich techniques, including transcriptomics, proteomics, lipidomics, glycomics, and metabolomics. While indispensable for interrogating molecular effects in specific contexts, these techniques do not directly capture a cell’s integrated phenotypic response. An alternative approach is cytological profiling, which uses high-content microscopy to measure hundreds of image-derived features that describe the morphological and functional state of single cells across subcellular compartments, allowing phenotypic effects to be quantified at high throughput^1,2^. Cell Painting (CP) is a standardized implementation of cytological profiling that facilitates multiplexed fluorescent labeling of key cellular structures, enabling rich phenotypic descriptions across diverse experimental conditions^3–5^.

CP is used to perform unbiased genetic and chemical screens and has become an assay of choice for exploring mechanism of action (MoA - please refer to a list of abbreviations in our Supplementary Information) and target identification studies in drug library screens^6–9^, especially valuable when the target is complex, considered undruggable and/or multipart (polygenic)^10,11^. Identifying the target(s) or pathway(s) the drug is acting on and, therefore, exposing its mechanism of action is crucial for optimizing drug therapeutic outcomes, increasing efficacy safety, and tackling side effects. CP allows the detection of an immense range of cell phenotypes by its multiparametric approach but becomes an even more useful technique when coupled with guilt-by-association studies. Although discovering the unique cell phenotype upon drug treatment could be exciting, similarities between cell phenotypes treated with different drugs can quickly enhance understanding of the effect of unknown drugs based on similarity to the drug with known MoA. In such an approach, it is standard to use the reference library of drugs with known MoA^1,2,12^. The bigger and more diverse a reference library is, the better the chance of finding the fingerprint profile of cell features extracted from images similar to the phenotype of interest.

Although Cell Painting is designed as an unbiased, high-content profiling method, it is typically performed on cells maintained in standard culture conditions, where environmental cues, tissue-specific signals, and intercellular interactions are absent. This limitation restricts the assay to the phenotypic landscape of resting cells, in which many signaling pathways remain inactive. As a result, many bioactive compounds fail to produce a detectable phenotype in standard CP screens—particularly when their targets are either not expressed or are functionally silent in the unperturbed state. These limitations give rise to a phenotypic “dark space”, in which the mechanisms of action for a substantial fraction of compounds remain obscured.

We hypothesized that inducing a defined activation state in cells would expand the range of observable phenotypes and help illuminate this dark space. We leveraged a similar approach in macrophages (RAW264.7), where lipopolysaccharide (LPS)-induced activation enabled the discovery of anti-inflammatory compounds by CP^13^. Here, we extend this strategy to A549 adenocarcinoma epithelial cells, a widely used model in cancer biology and drug discovery. A549 cells are also a standard in Cell Painting assays, with extensive reference datasets available for benchmarking^14,15^.

In the present study, we profile ~8500 known bioactive compounds at two concentrations (1 and 10 µM) to balance sensitivity with cytotoxicity. We perform CP on cells in both resting and activated states, the latter induced by 4-h treatment with phorbol 12-myristate 13-acetate (PMA), a potent protein kinase C (PKC) agonist that broadly stimulates signaling pathways controlling proliferation, differentiation, apoptosis, and immune response^16–18^. Due to its extensive impact on cellular signaling and morphology, PMA is ideally suited as a global activator in high-throughput screens. Here we demonstrate that cell activation with PMA reveals more phenotypes and enables the functional annotation of compounds that are otherwise inactive or phenotypically dark under resting cellular conditions.

## Results

### Drug-exposed A549 cells show the most distinguishable phenotypes in CP when activated with PMA for 4 h and stained according to the JUMP Consortium’s CP protocol 3.0

In preparation for high-throughput screening (HTS) of a large compound library, we aimed to establish robust cell-based assay conditions that would allow us to observe distinguishable phenotypes with high confidence. To achieve this, we tested the effects of two broad-spectrum activators on A549 cells: phorbol 12-myristate 13-acetate (PMA) at concentrations of 50 nM and 100 nM and Epidermal Growth Factor (EGF) at 50 ng/mL. These treatments were applied over three different incubation times − 28 h (dosed simultaneously with compounds), 18 h, and 4 h incubation time before cell fixing, staining and imaging.

**Fig. 1:**
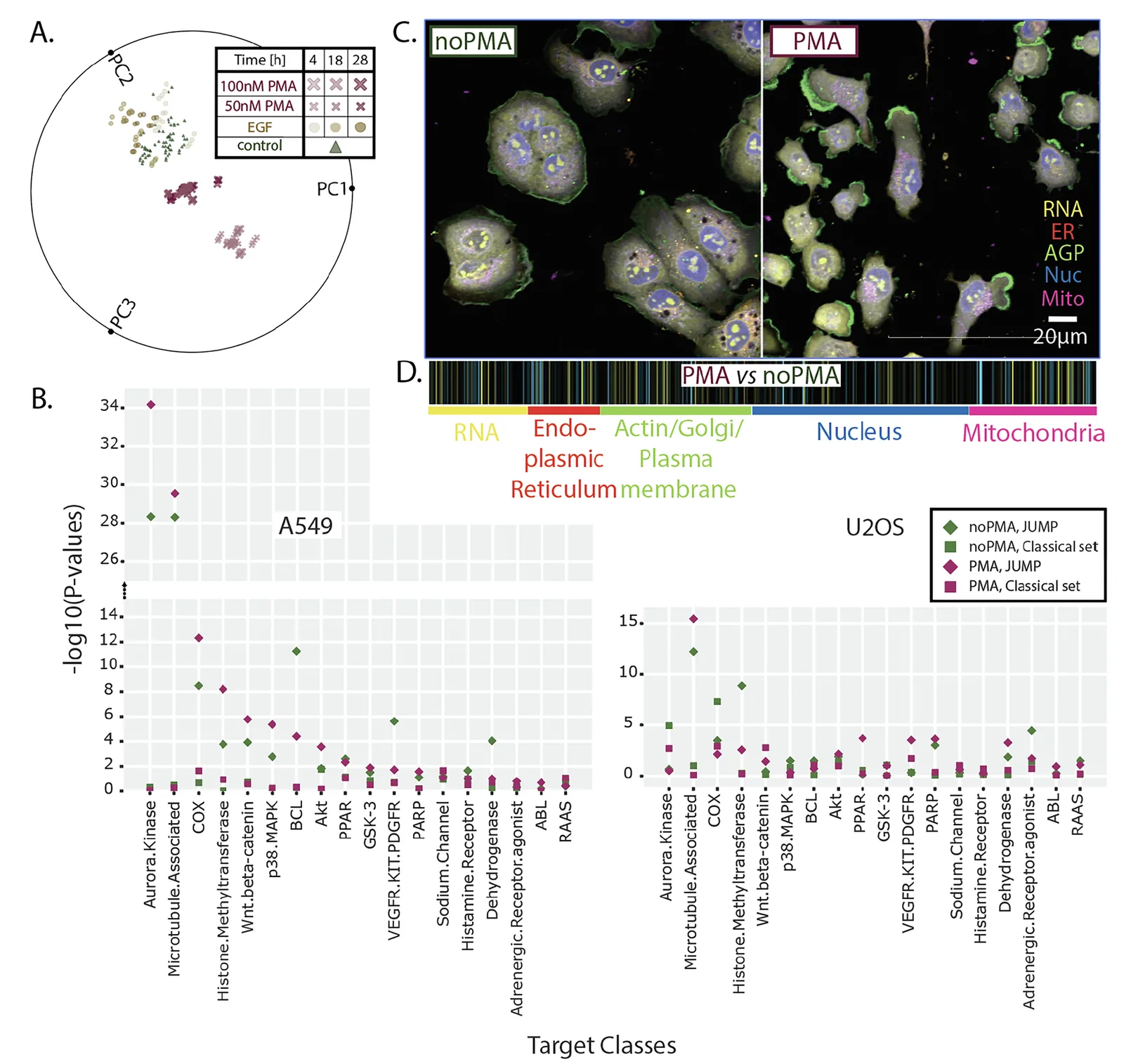
Cell activation and staining optimizations. Pilot data from A549 or U2OS cells treated with compounds, either rested (green), or activated with PMA (maroon) or EGF (gold) for 4, 18, or 28 h (indicated by marker brightness) and at two concentrations (indicated by marker size), stained with our classical stain set (squares, circles, triangles, or X) or Cell Painting stain set (used by the JUMP consortium, diamonds), performed in triplicate, and their phenotypes measured from microscopy images. **A** Radial plot of three principal components of cellular phenotypes for multiple replicate wells for each condition, all without compound and stained with the classical stain set. **B** Scatter plot from the K-S test, in which a higher -log(*p*-value) indicates that a particular target class is more readily distinguishable from other classes under the same condition. **C** Representative images of A549 cells without compound, with or without activation by 50 nM PMA for 4 h, stained with the Cell Painting stain set. Merged image shown have pseudocolor signal range maximum reduced to be visible (constant across all images shown). These images are representative of 672 independent experiments performed during the large-scale screen. **D** A fingerprint composed of 477 cellular features, showing how the mean across all wells increased (yellow) or decreased (cyan) upon activation with 50 nM PMA for 4 h. This was for the same conditions as (**C**).

Activation of cells with PMA for 4 h resulted in the most distinct phenotype (Fig.1A), as compared with control (untreated cells), EGF treatment, and other time points. The 100 nM concentration did not provide any additional benefit over 50 nM PMA, therefore, we selected 50 nM PMA for further testing.

A subsequent pilot assay determined the cell line and stain set leading to the best target class separation. In our previous CP in HeLa cells^19^, some compound target classes gave rise to easily distinguishable signatures, while others were more challenging, as judged by a K-S test (see methods for details). We therefore developed a panel of trial compounds that covered both types of target classes (9 high-scoring classes and 9 medium-scorers, with 6 compounds per class, Supplementary Table S2). This panel was used in PMA-activated and resting conditions, to compare two widely-used cell lines, A549 (adenocarcinoma epithelial cells) and U2OS (osteosarcoma cell line with epithelial morphology), and to compare two sets of dyes: the widely-used Cell Painting stain set optimized for cost, time efficiency, and ease of use by the JUMP Consortium (version 3)^14^ and our in-house classical stain set, which labels DNA, the Golgi apparatus, F-actin, nuclei, microtubules, mitochondria, and an inflammation marker, nitric oxide synthase (anti-iNOS), as described previously (Supplementary Table S5)^13^.

We observed the best performance with the Cell Painting stain set in A549 cells, where 69% of target classes showed significant in-class vs. out-of-class enrichment (Fig. 1B). The Cell Painting stain set showed significant enrichment (*p* < 0.05) for 62% of treatments, whereas our classical stain set distinguished only 12% of classes. For the U2OS cell line, 55% of the target classes showed significant enrichment in the Cell Painting stain set and 22% were enriched significantly in the classical stain set. This study showed that the cell lines are not agnostic with respect to the stain set used. Careful pilot studies should be performed to ensure the best cell line/stain set combination, as some combinations perform very poorly: for example, A549 cells using the classical stain set showed only 2% of classes scoring above the *p* < 0.05 threshold (Fig. 1B).

Furthermore, we observed that the 4-h, 50 nM PMA treatment affects cells very broadly (Fig. 1C), highlighting cellular features from all stained cell structures (Fig. 1D), confirming PMA as an appropriate global cell activator triggering multiple cellular pathways.

### Cell activation expands the phenotypic space illuminated by Cell Painting

**Fig. 2:**
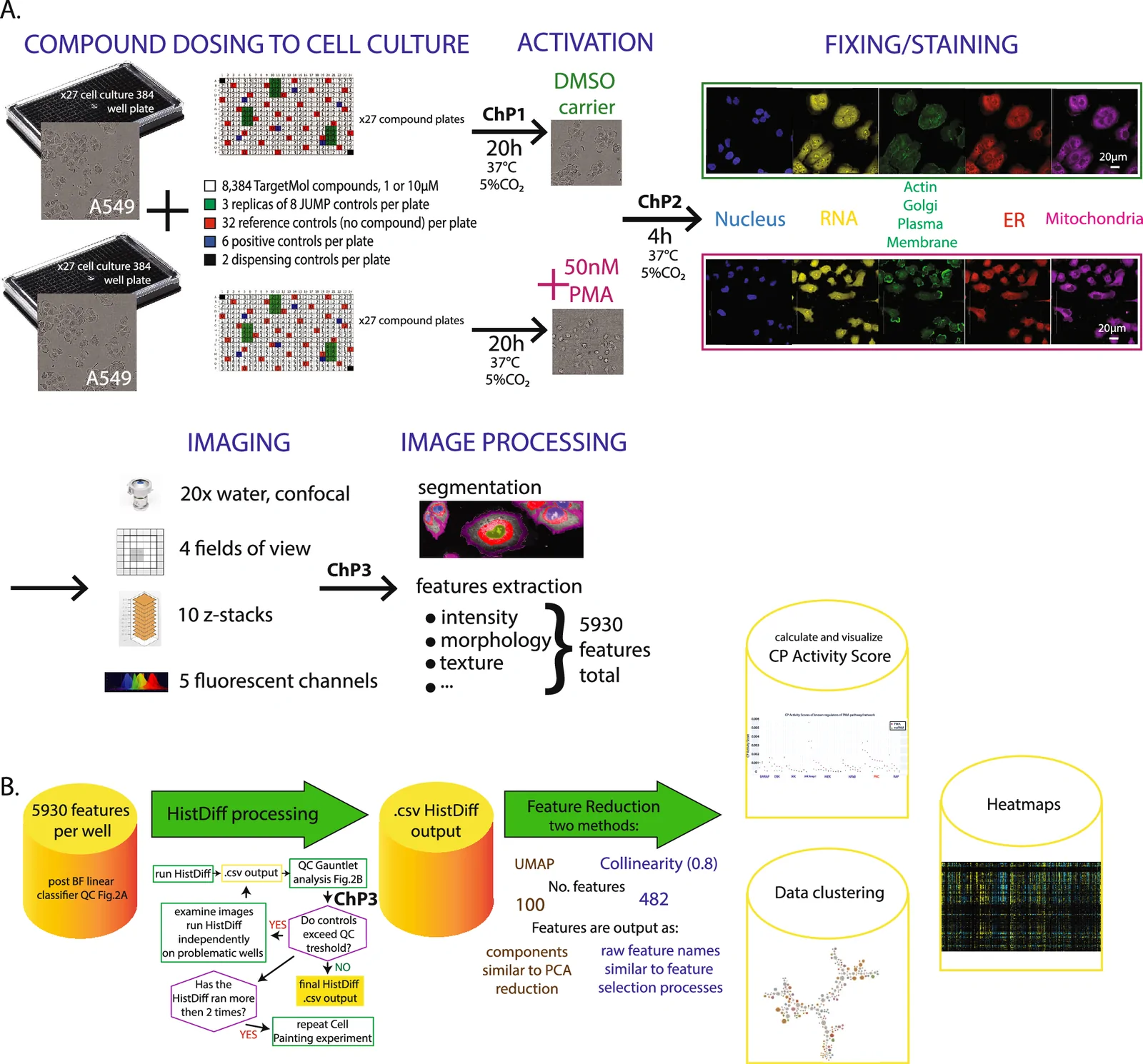
Screen workflow. **A** A549 cells were seeded, viewed at checkpoint 1 (ChP1), treated with 1 or 10 µM compounds using the plate map, either rested or PMA-activated, analyzed with an image linear classifier at checkpoint 2, stained, imaged, tiled into max-projected, merged montages at checkpoint 3, and quantified for cellular features. Microscopy images in A have pseudocolor signal range maximum reduced to be visible (constant across all images shown). **B** Single-cell feature data from individual wells were compared with the reference population in the same plate using HistDiff and visualized with QCGauntlet at checkpoint 4. The number of features was reduced, using UMAP or collinearity, and clustered. CP Activity Scores were calculated and heatmaps produced from collinear-reduced features.

We screened a diverse library of 8387 bioactives (TargetMol L4000-CUST), representing 815 drug classes, at 1 and 10 µM in A549 cells with a 24-h incubation, either with or without PMA activation (Fig. 2A). The resulting phenotypic fingerprints consist of hundreds of cell features extracted from the segmented regions of the images for each cell and further processed using the HistDiff algorithm, which normalizes each feature vs. the collected DMSO vehicle controls from the same plate (Fig. 2B)^2^.

As this chemical library was tested in two drug concentrations (1 and 10 µM) in resting and PMA-activated cells, and several plates were tested in replicas for quality control (QC) reasons, the combined number of conditions tested sum up to almost 50,000 individual wells. At this scale, obtaining technical replicates of each compound was cost-prohibitive, prompting the development of a rigorous quality control pipeline to ensure reproducibility.

Each plate contained 38 vehicle-only control wells (including a combination of DMSO-only and PMA-only wells in both the PMA-treated and untreated plates) in randomized positions (Fig. 2A), providing extensive within-plate references for normalization and quality assessment. All controls were distributed within each plate using a mixed integer linear program (MILP) designed specifically to minimize positional and batch effects. The noPMA and PMA conditions were processed in parallel batches with identical plate maps, ensuring that any given compound occupied the same position in the two conditions, thereby controlling for systematic positional effects between conditions. We designed and implemented additional advanced quality control methods to confirm activation and verify reproducibility among the activation and positive controls, as described in the results section and Supplementary Fig. S1 of the Supplementary Information.

**Fig. 3:**
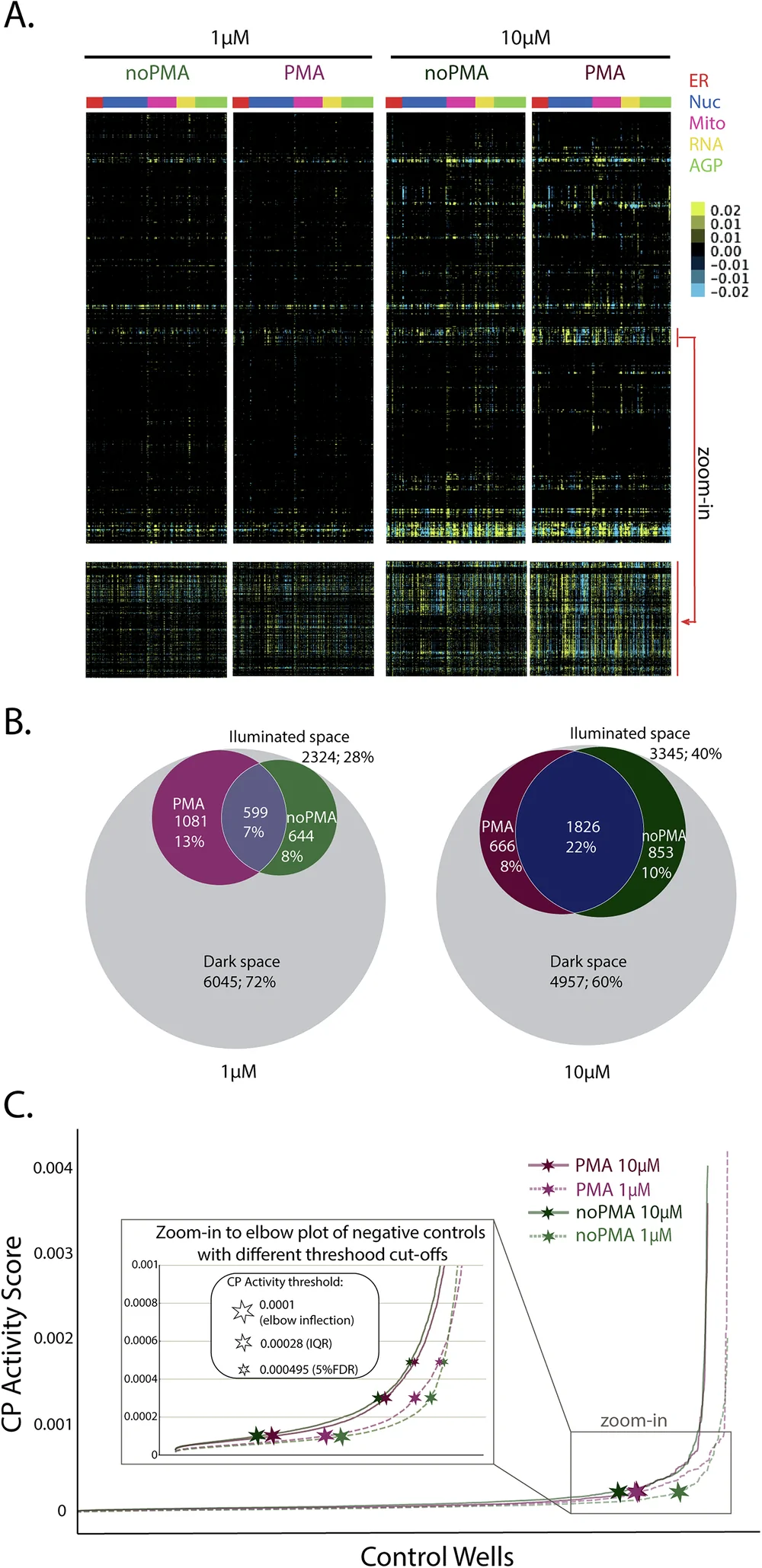
Visualizations of the entire screen dataset. **A** Heatmaps of the phenotypic fingerprints (*x-axis*), each a combination of cell features associated with 5 imaged channels (ER, nuclei, mitochondria, RNA, or actin-Golgi-plasma membrane, as indicated at the top) that were increased (yellow), decreased (cyan), or unchanged (black) compared to no-compound reference, for all 8387 compounds tested (*y-axis*), at 1 (left) or 10 µM (right), in resting (green, left) or PMA-activated (maroon, right) cells. In this visualization, compounds are sorted according to clustering in the 10 µM with PMA dataset (rightmost heatmap). The red line points to a zoomed-in section of ∼350 compounds (bottom). **B** Venn diagrams of all of the compounds tested (largest circle), showing how many, semi colon, and what percent of compounds had no significant phenotypic differences (gray) compared to no-compound reference controls, a phenotype only in the resting cells (green), a phenotype only in the PMA-activated cells (maroon), or a phenotype in both conditions (violet). The cutoff for a positive phenotype in this analysis was the IQR CP Activity Score threshold, at which a quarter of the reference controls (i.e. outliers) would be assigned as positive. **C** Elbow plot of the CP Activity Scores sorted in ascending order, for no-compound reference control wells, showing the cut-off for what is considered a positive phenotype, determined 3 different ways, distinguishable on a zoomed-in inset: elbow method (large stars), IQR (medium stars), or 5% FDR, small stars).

The entire screen dataset (all doses, all conditions, all compounds) is visualized in Fig. 3A–C, enabling global comparison between resting and PMA-activated cells and between the high- and low-dose drug treatments. The fingerprints were clustered to generate heatmaps of compounds (*y-axis*) *vs*. cellular features (*x-axis*, separated by cellular compartment) (Fig. 3A). As expected, drugs dosed at 10 µM effected more dramatic phenotypic changes in cells compared to the 1-µM treatment, as observed on the global and zoomed-in sections of the heatmap (Fig. 3A). However, the higher dose also led to a 2-fold increase in the number of cytotoxic compounds (Supplementary Fig. S2C).

To assess the impact of our cell activation platform, we sought to evaluate the benefit of illuminating additional phenotypes versus the cost of doubling the screen size. To do this, we measured the size of the phenotypic space covered by each condition (resting or PMA-activated, 1 or 10 µM). To do this, first we calculated the CP Activity Score, which reflects the cumulative effect of treatment on cells and its magnitude. Higher CP Activity Score values indicate more features differing more dramatically from no-compound controls, indicating a more robust phenotype. We then selected a normalized CP Activity score threshold for distinguishing bioactive from inactive compounds, defining the inactive (dark) space using the combined 3404 no-compound control wells in the screen (Fig. 2A, red wells). We used the interquartile range (IQR) method to establish the activity threshold (Fig. 3C, medium-sized stars), in which the outliers above the 75th percentile were excluded since they do not fit the general distribution. Through this approach, we determined a normalized CP Activity Score for the cut-off as 0.00028.

Using this threshold, when comparing the different drug concentrations, we could see that 28% and 40% of all screened compounds, in 1 µM and 10 µM dosing, respectively, produced an illuminated effect on cell phenotype (Fig. 3B). This increase in illuminated space is consistent with what we would expect, since higher doses of compound should give more pronounced effects on cells, including more cytotoxic and off-target effects (i.e. a potentially less informative effect). When comparing PMA-activated versus resting cells, as expected, we find that there are some compounds that are illuminated no matter the cell state: 7% of compounds at 1 µM and 22% of compounds at 10 µM dosing (Fig. 3B, violet shading).

The most interesting compounds are those that were only illuminated in one of the two conditions - some were specific to resting cells (Fig. 3B, green), and some were specific to PMA-activated cells (Fig. 3B, maroon). This confirms that adding cell activation to the screen identifies additional phenotypic space. In other words, 13% of all screened compounds would stay in the dark space at 1 µM if the assay did not include PMA activation; this means we now have insight into the MoA for more than 1000 drugs.

We also present alternate methods for selecting the activity threshold (Fig. 3C and Supplementary Fig. S2A, B). We found that the illumination rate varied wildly, from 16.1 to 80.9%, depending on the different thresholds, as well as the concentrations used in a screen. In these cases, the benefit of using PMA can reach as high as 22.5% of the compounds now illuminated (which is 54% added value; Supplementary Fig. S2B, light maroon).

### PMA activation reveals latent pathway structure through target-class consolidation and fragmentation

**Fig. 4:**
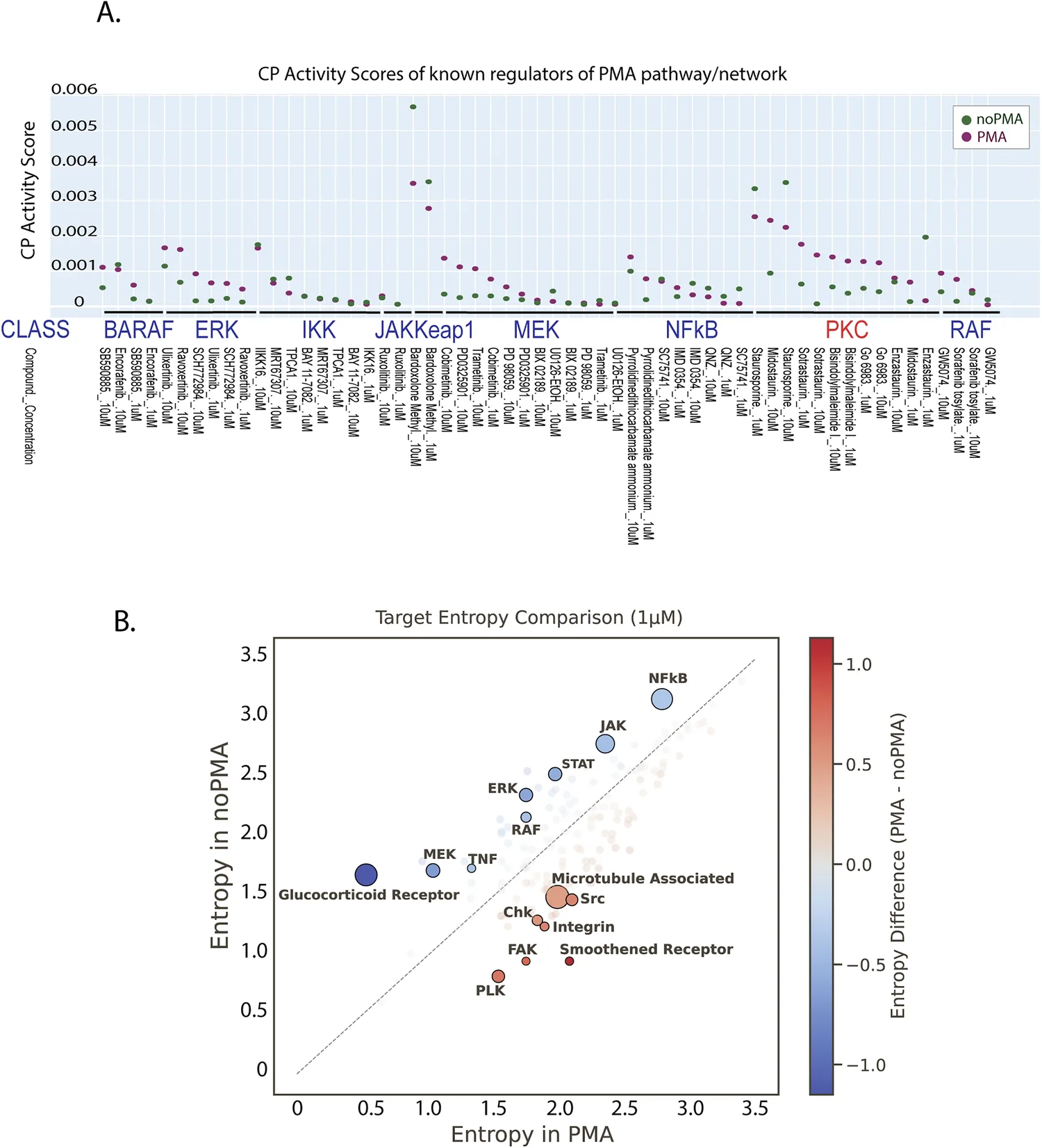
Target classes. **A** Scatter plot of the CP Activity Scores in resting cells (green) or PMA-activated cells (maroon) for compounds modulating the direct target of PMA (PKC - in red) or 8 other targets in the network (blue). **B** An entropy analysis of how similar the phenotypes are between members of a target class. where the marker size correlates to the number of compounds in that class (e.g. FAK has 7 compounds and microtubule associated has 50 compounds), and color indicates whether the phenotypes within the class became more (blue) or less (red) similar to PMA activation.

To validate our results, we looked at the direct target of PMA, which is protein kinase C (PKC). This enzyme plays a critical role in signal transduction pathways and regulates cell growth and differentiation^18^, PMA exerts its effect on distinct cell metabolic areas through an intricate network of affected pathways. Indeed, thanks to PMA activation, we can clearly observe the phenotypic effect of PKC, MEK, and ERK inhibitors in PMA-treated cells, among others, giving us confidence in the screen results (Fig.4A).

To understand how acute pathway activation alters the functional relationships between compound classes, we examined shifts in phenotypic clustering of inhibitors within their annotated classes in response to PMA activation. Using the pairwise Euclidean distances between the CP fingerprints of the active compounds (using the 0.0001-CP score threshold, Fig. 3C and Supplementary Fig. S2B) at 1 µM, we performed entropy analysis to compare their in-class vs. out-of-class distances in the resting vs. PMA-activated condition. This analysis is described in the supplementary methods section of Supplementary Information.

This analysis revealed that several target classes, including the glucocorticoid receptor (GR), MAPK pathway components (RAF, MEK, ERK), and JAK/STAT signaling proteins, became markedly more consolidated, that is, they clustered more tightly within their annotated class, upon PMA treatment (Fig. 4B, blue). For example, GR-targeting compounds, which displayed more heterogeneous phenotypes in resting cells, clustered tightly in the presence of PMA, consistent with literature describing antagonism between GR signaling and PKC-induced transcription factors such as AP-1 and NF-kappaB^21,22^. Similarly, inhibitors of RAF, MEK, and ERK coalesced into a shared phenotypic space under PKC activation, likely reflecting their common suppression of the PKC -> RAS -> RAF - > MEK - > ERK cascade, which is robustly activated by PMA^16^. These observations suggest that PKC activation not only amplifies specific signaling pathways but also synchronizes the phenotypic effects of compounds acting on those pathways, effectively identifying latent pathway-level relationships.

In contrast, other target classes became less consolidated under PMA treatment (Fig. 4B, red), reflecting greater phenotypic diversity upon PKC activation. Notably, PLK inhibitors, which clustered tightly in the no-PMA condition—likely due to a shared mitotic arrest phenotype—exhibited increased entropy and dispersed into multiple clusters in the activated state. This may result from PKC-induced cell cycle arrest or differentiation, which alters the cellular context in which mitotic kinases are inhibited^23^. A similar loss of consolidation was observed for focal adhesion regulators (e.g. FAK, integrin, Src), whose phenotypes may be modulated by PKC-dependent changes in cytoskeletal organization and adhesion dynamics^24^. Furthermore, the decrease in consolidation may reflect a lack of activity resulting from loss of the primary mechanisms-of-action of compounds that are present in the standard culture conditions, thus now amplifying more subtle off-target compound activities.

Together, these findings suggest that acute pathway activation reorganizes the phenotypic landscape, consolidating the phenotypes of compounds targeting convergent pathways, while dispersing the phenotypes of compounds whose activity becomes context-dependent under activation. This highlights the value of multi-point perturbation—such as combining functional compounds with activators—in identifying hidden structures in phenotypic response space and mapping conditional dependencies between signaling pathways.

### Glucocorticoid receptor agonistic activity predicted from screen network exploration is validated in cells for natural product, 2-methoxycinnamaldehyde

**Fig. 5:**
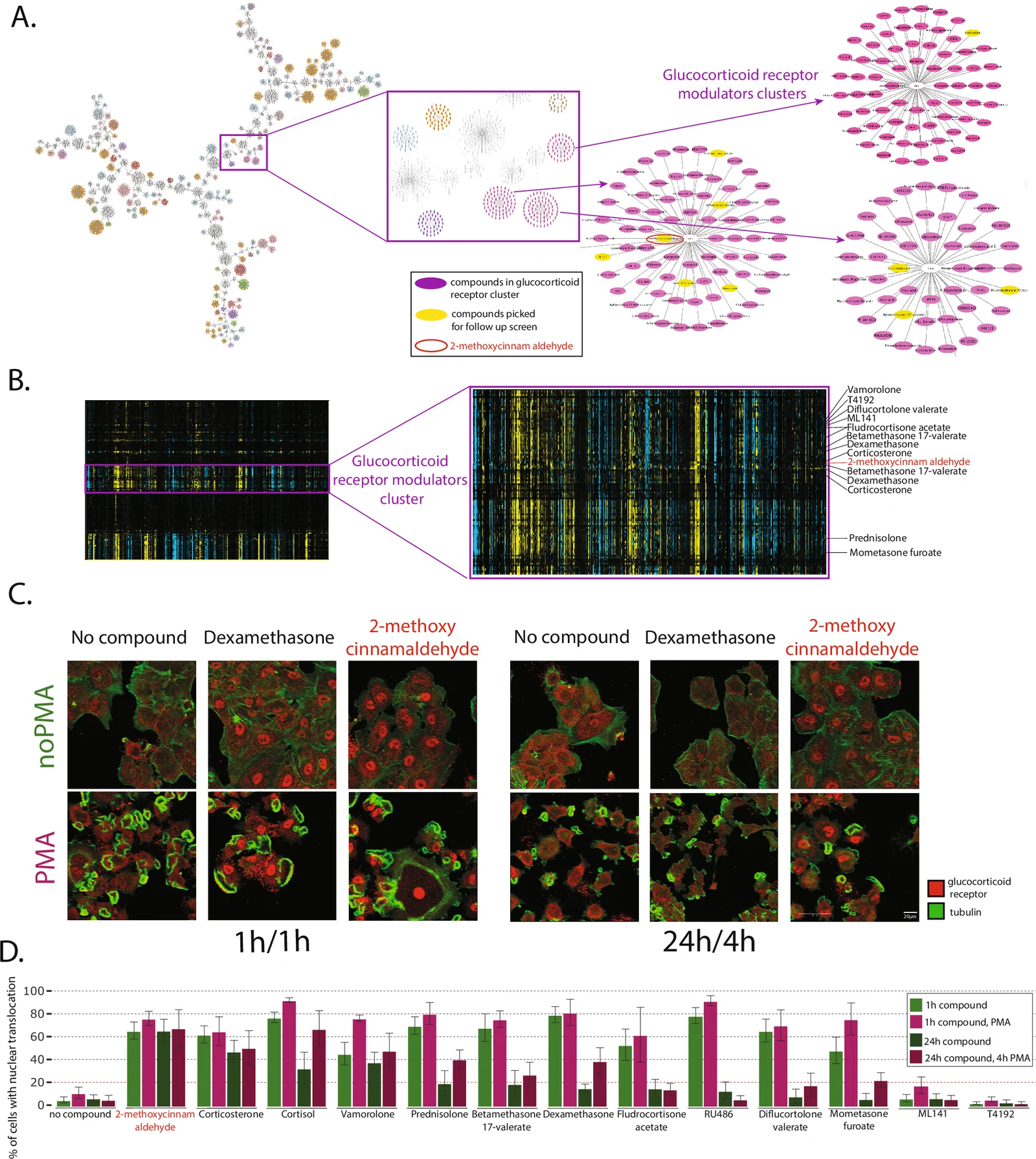
Cell phenotypes of 2-methoxycinnamaldehyde and glucocorticoid receptor (GR) ligands. **A** Compound similarity network using UMAP features for the entire screen (left), zoomed-in on the region of GR ligands (pink box, middle), and further zoomed-in on the 3 GR ligand clusters (right, 10 compounds chosen for follow-up in yellow). **B** The entire screen data visualized on a single heatmap (2-way clustered with features on *x-axis* and compounds on *y-axis*) and zoomed-in on the region of GR ligands (left) and further zoomed-in on compounds of interest (right) clustering near 2-methoxycinnamaldehyde (pink). **C** Representative 63× images of A549 cells treated with DMSO vehicle (no compound), 1 µM GR agonist dexamethasone, or 1 µM 2-methoxycinnamaldehyde for 1 h (left) or 24 h (right), followed by resting (top) or PMA-activation (bottom) for 1 h or 4 h, respectively, then fixed and stained for tubulin (green) and GR (red), with merged image pseudocolor signal range maximum reduced to be visible (constant across all images shown). **D** Nuclear translocation of GR in resting (green bars) or PMA-activated (maroon bars) cells treated with DMSO vehicle (left), 2-methoxycinnamaldehyde (pink), 10 known GR ligands, or 2 other compounds in the cluster. Data are presented as mean values +/− SD representing four fields of view for each of two separate wells.

Since compounds targeting GR showed the highest in-class clustering upon PMA treatment among the >800 annotated target classes in the library (Fig. 4B), we examined the compounds in this cluster more closely. Clustering among the known GR modulators were three compounds not annotated as associated with GR signaling: 2-methoxycinnamaldehyde (assigned to the apoptosis target class), ML141 (a Cdc42 modulator), and hnps-PLA inhibitor (a phospholipase inhibitor) (Fig. 5A, B). To test whether these compounds affect GR activity (directly or indirectly), we employed an immunofluorescence assay that tracks GR nuclear translocation, a hallmark of its activation^25^. The GR normally resides in the cytoplasm, but upon binding to agonists, the complex translocates to the nucleus, where it acts as a transcription factor. As positive controls, we used ten known GR regulators that were also found in this CP cluster. The nuclear translocation is fast and can be observed within 1 h. Because the original CP screen was done using a 24-h compound treatment, we examined the GR nuclear translocation at both 1-h and 24-h drug treatments.

We observed that 2-methoxycinnamaldehyde caused GR to translocate from the cytoplasm to the nucleus (Fig. 5C, red); this effect is clear at 1 h and persists at 24 h. 2-methoxycinnamaldehyde is a relatively simple natural product isolated from the plant genus *Cinnamomum* and in *Illicium verum {*https://lotus.naturalproducts.net/*} {LOTUS database}*), and has anti-inflammatory properties^26,27^, specifically an inhibitory effect on NF-kappaB^28^ and antioxidant potential^29^. We observed varied persistence of GR nuclear localization across the compounds in the cluster. Over 60% of cells treated with 2-methoxycinnamaldehyde retained high nuclear GR levels after 24 h, a proportion greater than for any other compound tested (Fig. 5D, dark green).

We also investigated the effect of PMA treatment on GR nuclear translocation in the presence of these compounds. Interestingly, activating cells with PMA enhanced drug-induced nuclear translocation of GR in almost all cases relative to the no-PMA condition. GR translocation was more pronounced upon PMA treatment at the 24-h time point, consistent with the increased consolidation (decreased entropy) of the GR modulators in the presence of PMA after 24 h. In the presence of PMA, after 24 h 2-methoxycinnamaldehyde was able to induce GR translocation to almost the same level as cortisol (Fig. 5D, maroon).

## Discussion

Cell Painting, or Cytological Profiling (CP), has emerged as a powerful technique for unbiased exploration of cellular phenotypes and drug mechanisms of action. However, the standard approach of profiling resting cells captures only a limited view of the cellular response landscape. In this study, we demonstrate that activating cells with PMA prior to CP analysis expands the observable phenotypic space and enables the detection of otherwise inactive compounds. By screening nearly 8500 bioactive compounds in both resting and activated states across two concentrations, we were able to illuminate an additional 13% of compounds that showed significant phenotypic effects only in the activated state at 1 µM, representing over 1000 compounds whose activities would have remained hidden in traditional CP screens.

The choice of activation conditions proved critical for maximizing the information content of our CP profiles. Based on our pilot studies with 72 compounds representing 18 different mechanistic classes, a systematic comparison of different activators, concentrations, and timing revealed that 4 h treatment with 50 nM PMA provided optimal cellular responses while maintaining cell viability. This finding exemplifies the importance of careful optimization of activation parameters, as too little stimulation fails to alter the phenotypic space, while excessive activation can mask compound effects. The broad impact of PMA on cellular signaling pathways, evidenced by our feature analysis showing effects across multiple cellular compartments, makes it particularly well-suited as an activator for broad screening approaches not directed towards a specific target pathway.

Our implementation of multiple quality control checkpoints, including a machine learning-based classifier for detecting cellular activation states quickly from bright-field images, allowed robust and reproducible screening results. The high reproducibility of our 8 positive controls across wells, plates, and batches validates our experimental approach while demonstrating how proper quality control measures can ensure reliable data generation in complex, high-content screens. The development of the QCGauntlet visualization tool further facilitates rapid assessment of plate-level data quality and compound effects, providing a useful quality control resource for the CP community.

The observation that PMA activation drives consolidation of certain target classes while fragmenting others highlights the dynamic interplay between signaling context and compound activity^30^. Pathways such as GR signaling and the MAPK cascade (RAF, MEK, ERK) exhibited tighter clustering upon PKC activation, suggesting that phenotypic responses within these pathways become more uniform when they are strongly engaged or modulated. This is consistent with known antagonistic interactions between PKC-driven transcription factors (such as AP-1 and NF-kappaB) and the GR, where activation of one pathway constrains or homogenizes the functional effects of the other^21^. Similarly, the unification of MAPK inhibitors under PMA likely reflects convergence on the RAS -> RAF -> MEK -> ERK axis downstream of PKC, where inhibition of any node in the pathway blocks a common signaling output^16^. These results suggest that strong pathway activation can amplify otherwise subtle pharmacologic effects, revealing coherent drug response clusters that might remain hidden under basal conditions.

Conversely, the fragmentation of target classes such as PLK inhibitors, microtubule poisons, and focal adhesion regulators suggests that PKC activation can also introduce heterogeneity, depending on the biological context. PKC-driven changes in cell cycle progression^23^, adhesion dynamics^30^, or cytoskeletal organization likely create dynamic cellular states in which the activities of these inhibitors become more variable. In particular, PLK inhibitors, which induce a stereotyped mitotic arrest in proliferating cells^31,32^, may act less uniformly in cells that have exited the cell cycle or undergone PKC-induced differentiation. Thus, pathway activation not only consolidates phenotypic responses for some target classes but also unmasks biological complexity for others. These findings emphasize the importance of studying compound effects across multiple cellular states to fully capture context-dependent mechanisms of action and uncover latent functional connectivity between pathways.

The biological relevance of activation-dependent phenotypes is demonstrated by our discovery of GR translocation upon treatment with 2-methoxycinnamaldehyde. This natural product, previously known for its anti-inflammatory properties, was found to cluster with known GR modulators in our phenotypic analysis, leading to experimental validation of its ability to induce GR nuclear translocation. Notably, this effect was enhanced in PMA-activated cells, suggesting that cellular activation can reveal both compound activities and potential synergistic effects. The persistent nuclear localization of GR, even after 24 h, upon treatment with 2-methoxycinnamaldehyde, indicates a sustained effect that distinguishes this compound from a panel of classical GR modulators. The presence of an electrophilic aldehyde moiety in this simple compound may indicate that its cellular activity arises from covalent modification of its target, which would be consistent with its prolonged effect on GR nuclear localization.

Cell Painting data are inherently noisy, arising from biological variability at the single-cell level, staining heterogeneity, and positional effects within and across plates. Technical replicates are therefore valuable for distinguishing true compound-induced phenotypes from stochastic variation. A key limitation of this study is that individual compound treatments were not replicated. Screening ~8500 compounds at two concentrations in both resting and PMA-activated states already required nearly 50,000 wells; incorporating even duplicate measurements would have doubled the screen size, making it cost-prohibitive. To mitigate this, we employed 38 randomized vehicle-only control wells per plate, a MILP-optimized plate layout to minimize positional and batch effects, identical plate maps across conditions, and a multi-checkpoint quality control pipeline including a machine learning-based activation classifier and the QCGauntlet visualization tool. While these measures provide confidence in the reproducibility of our data, the absence of technical replicates means that the activity of any individual compound should be validated independently before drawing mechanistic conclusions, as we demonstrated for 2-methoxycinnamaldehyde and the GR cluster.

The decision to screen compounds at both 1 µM and 10 µM concentrations provided complementary insights into compound activities. While the higher concentration revealed more dramatic phenotypic changes and enabled the detection of weakly active compounds, the lower concentration helped identify more specific and potentially physiologically relevant effects while minimizing cytotoxicity. This dual-concentration approach, combined with cell activation, creates a more comprehensive view of compound activities across a broader dynamic range than traditional single-concentration screens in resting cells.

Our findings also emphasize the importance of proper feature selection and data analysis strategies in CP. The comparison of different feature reduction methods - UMAP for similarity-based clustering versus collinearity-based selection for mechanistic interpretation - demonstrates how different analytical approaches can extract complementary insights from the same underlying data. The success of our clustering approach in identifying compounds with similar mechanisms of action, as validated by the GR example, supports the utility of guilt-by-association analyses in activated cells for mechanism of action studies.

These results establish activated CP as a useful approach for expanding the observable phenotypic space in high-content screening. The significant increase in detectable compound activities, combined with the discovery of mechanisms for known compounds, suggests that many bioactive molecules may have additional activities that are only revealed in activated cellular states. This work provides a methodological approach and analytical tools for implementing activated CP screens while deepening our understanding of the importance of cellular context in small molecule profiling. Future studies may further expand this approach by exploring different activators and cell types to illuminate additional aspects of compound-cell interactions.

## Methods

### Chemicals

We obtained a library of 2035 bioactive compounds (L1700, listed in Supplementary Table S2) from SelleckChem, which were used in the pilot. We obtained a library of 8387 bioactive compounds (L4000-CUST, listed in Supplementary Data 1) from TargetMol. These were all the known bioactives available in solution format from TargetMol at the time of purchase by CSC (2022-05-03). Daughter plates of the TargetMol library (27 384-well Labcyte PP0200 plates) were prepared as 2.5 mM stocks in 90% DMSO, and were used in our CP screen and GR assay. All library daughter plates received food coloring dye in well A1 (for a visual confirmation of Echo transfer step). All library daughter plates also received 8 positive control compounds, as proposed by the JUMP Consortium^9^ with modification (Supplementary Table S3). These libraries are housed at the UCSC Chemical Screening Center (RRID:SCR_021114), and more information is available at {https://csc.ucsc.edu/compounds/} {UCSC CSC website}.

### Cell culture

A549 (ATCC Cat. CRM-CCL-185) and U2OS (ATCC Cat. HTB-96) were cultured in DMEM media (Fisher, Cat. 21063029), plus 10% FBS (Fisher, Cat. NC0961964) and 1× penn strep at 37 °C, 5% CO_2_, 95% humidity. For the pilot, CP screen, and GR assay, cells were seeded at 1250 cells/well in 25 µl by peristaltic bulk dispenser BioTek EL406. For the pilot, we used 384-well Corning 3764 plates, and for the CP screen and GR assay, we used PhenoPlates (Perkin Elmer, Cat. 6057300).

### Drug treatment

All compounds and activators were dosed by Labcyte Echo 650 (Beckman Coulter, UCSC Chemical Screening Center, RRID:SCR_021114) and Plate Reformat software v1.8, using Labcyte 384PP (Cat. 001-14555) or 384LDV (Cat. 001-12782) source plates. Well A1 was used to transfer food coloring for a visual confirmation of having run the Echo step.

For the pilot, A549 and U2OS cells were treated with 108 bioactives from SelleckChem at 10 µM for 28 h. In the CP screen, A549 cells were treated with the 8387 bioactives from TargetMol. Each 384-well assay plate had 320 wells that were treated with bioactives for 24 h, and the remaining 64 wells were used for various controls. The entire screen was conducted twice, with compound dosing at 1 or 10 µM. In the 1 µM CP screen, all 64 no-compound control wells received vehicle treatment from wells containing 90% DMSO. In the 10 µM CP screen, eight JUMP control compounds (Supplementary Table S3) were each used in triplicate on each plate (i.e. 24 of the 64 control wells on every plate were used for these JUMP positive control compounds - see green wells in the plate map on Fig. 2A). The remaining 40 no-compound control wells in the 10 µM CP screen received vehicle treatment from wells containing 90% DMSO (see red and blue wells in the plate map on Fig. 2A), and were used as the reference signal for most analyses.

For screening, the 62 control wells (which include no-compound reference controls, blocks of JUMP controls, and cell activation controls) were placed in randomized locations on the assay destination plates, using either the JUMP plate map (for our 1 µM CP screen) or a custom randomized plate map developed by Benjamin David and Assistant Professor Paul Jensen, at the University of Michigan Department of Biomedical Engineering using a mixed integer linear program (MILP) performed with a custom design software implemented in Julia version 1.8.2. The exceptions to the randomization strategy were dispensing controls: wells A01 and P24 reserved for a dye, for visual QC of Echo transfer.

For the GR assay, cells were treated for 1 h or 24 h with 1 µM hit compounds (2-methoxycinnamaldehyde, ML14, or T4192) or 10 known GR agonists (RU486, prednisolone, mometasone furoate, dexamethasone, cortisol, vamorolone, fludrocortisone acetate, diflucortolone valerate, corticosterone, or betamethasone 17-valerate), from the TargetMol library (product #L4000-CUST) with 2 well replicates of each condition. On each plate, the remaining wells were treated with a DMSO vehicle. There were 64 such wells in the 1 µM screen and 40 wells in the 10 µM screen.

### Cell activation

After drug treatment, the cells were activated using the Echo 650. For the pilot, cells received 50 or 100 nM PMA (Fisher, Cat. AC356150010) from a stock in 90% DMSO or 50 ng/ml EGF (VWR International LLC, Cat. 10781-692) from a stock in water. They were activated either immediately after drug treatment (which was 28 h before fixing), 18 h, or 4 h before fixing.

For the CP screen, 4 h incubation with 50 nM PMA was used. The entire CP screen was conducted twice, such that every treatment (i.e. compound and dose) was performed with cells that were PMA-activated as well as resting cells that received no PMA activation (therefore, there were 108 assay plates for screening 27 compound plates). For the 10 µM CP screen, well P24 was used at the activation step to transfer food coloring for a visual confirmation of having run the Echo step.

For the GR assay, 1 h or 4 h incubation with 50 nM PMA was used: in one set of cells, activation and compound treatment both started 1 h before fixing, while another set of cells were treated with compound for 24 h, with the final 4 h of this incubation being in the presence of PMA.

### Quality control (QC) ChP2: verify activation by a linear classifier

We used an imaging-based, machine-learning linear classifier method to verify that live cells were activated by PMA, before proceeding to fix and stain the cells (for results of this, see supplemental information). 2 h after PMA treatment (22 h after compound treatment), the entire batch of plates of live cells was moved to random access racks in a LiCONiC StoreX Precision incubator at 37 °C with 5% CO_2_. The plates were individually transferred between the incubator and the environmental chamber of the microscope using a PreciseFlex P(3)400 robot with a gripper using Plate::Works v6.25 scheduling software. A single field of view for each control well was imaged on a Revvity Opera Phenix Plus with a 10× air objective, two peak autofocusing, exposure time of 2 ms, transmitted light power at 30%, in widefield mode, and with binning of 2.

An analysis was run online concurrently with imaging in Harmony 5.1 software. The images underwent brightfield correction (a variation of flatfield correction for uneven illumination at the corners), the corrected image was smoothed (using a median filter with a 2 px scale), and the smoothed image was then inverted (using a 60% quantile cut-off). The find cells module was run on this inverted image using method B, with a common threshold of 0.21, predicted area >100/µm^2^, splitting coefficient of 11.6, an individual threshold of 0.16, and contrast ratio >0.04. The cells were then filtered to remove the border objects. For each cell, we calculated 6 standard morphology properties (area, roundness, perimeter, width, length, width to length ratio), brightfield STAR morphology properties (symmetry, threshold compactness, axial, and radial, with profile (4 px), and sliding parabola (curvature of 10), 9 standard brightfield intensity (mean, standard deviation, coefficient of variance, median, sum, maximum, minimum, quantile fraction at 50%, and contrast), and 8 brightfield texture SER properties (scale 0 px and normalized by kernel to locate spot, hole, edge, ridge, valley, saddle, bright, and dark features). Putative cells that were too big (area >2000/µm^2^) or too dark (brightfield mean <10,000) were removed from consideration.

A linear classifier-based analysis was determined using the training data from a single plate. All the cells in two random resting (no PMA) wells and all the cells in two random PMA-activated wells were the training set of cells for two phenotypes (taking into consideration all the 23 measured features described above for each cell). The goodness of the model was 1.05, with an offset of −1.188. The distinguishing properties selected by machine learning in the final trained model were three texture measurements (dark, valley, and edge) and four intensity measurements (mean, median, contrast, and maximum). This pre-trained classifier was then applied to all cells in all wells of all future plates. In each plate analyzed, the percent activated is calculated (100*a/(a + b) where a is the number of cells that appear activated, and b is the number of cells that appear to have resting phenotype). The percent activated was reported as a bar graph of stimulation (Supplementary Fig. S1A) for each plate. This analysis was performed on all plates from the 10 µM screen. All plates tested passed quality control by the linear classifier, with the criterion that they have no more than two control wells (<10% of the wells) with greater than 15% of the cells therein classified as activated in noPMA wells and have no more than two control wells with less than 30% of the cells classify as activated in the PMA wells.

### Staining

All fixing and staining steps were conducted on a BioTek EL406 device equipped with a 10 or 5 μl peristaltic pump cassette and a 192-tube wash module. For the pilot, three stain sets were used on separate plates. Two of the sets, together, are called the classical set herein, were stained as described in Lau et al. ^13^. The third set in the pilot and the screen were stained as described by the JUMP Consortium^14^ using their Cell Painting stains (designated the Cell Painting or JUMP stain set) and protocol version 3 (with one assay modification: the drug incubation time was reduced from 48 h to 24 h to limit drug secondary effects). The GR assay was stained with three different stain combinations on one plate. The list of all stains used is available in Supplementary Table S4. The staining steps are outlined in Supplementary Table S5.

### Imaging

For all imaging in this paper, four fields of view for all 384 wells of the plate were imaged on a Revvity (called Perkin Elmer at the time of manufacture) Opera Phenix Plus (UCSC Chemical Screening Center, RRID:SCR_021114) with a 20×/1.0 NA water objective, two peak autofocusing, in confocal mode with a 50 µm pinhole spinning disk, two simultaneous 2160 × 2160px cameras, and with binning of 2. A 10-plane Z-stack was collected with 0.8 µm between planes (7.2 µm sample height covered in total). Supplementary Table S6 shows a list of channel wavelengths, their laser powers, exposure times, and channel orders for 2-camera simultaneous capture pairings used in imaging.

For the screen, the entire batch of fixed, stained cell assay plates was moved to a random-access plate hotel at room temperature. The plates were individually transferred between the rack and the microscope using a PreciseFlex P(3)400 robot with a gripper, using Plate::Works v6.25 scheduling software. A data transfer to the Signals Image Artist v1.1 server was run online concurrently with imaging in Harmony 5.1 software.

### Image quantification

For the pilot and the CP screen, image analysis was run using the Signals Image Artist v1.1 web app connected to our Linux data storage server. The images underwent advanced flat-field correction and max projection of the Z-stack. For the 10 µM screen, the images also underwent stitching to create a global image, combining all fields of view with dynamic binning. The find nuclei module was run on this corrected, max-projected (and stitched in the case of 10 µM) blue channel image using method B, with a common threshold of 0.62, predicted area >30 µ^2^, splitting coefficient of 7.0, individual threshold of 0.4, and contrast ratio >0.1. The find cytoplasm module was then run on the associated red channel image to find cell regions surrounding the nuclei from the previous step. It used method B, with a common threshold of 0.45 and an individual threshold of 0.15. In the case of the 10 µM screen data, the cells were then filtered to remove the border objects. For each cell in each well, we divided the cytoplasm into three sections: the outermost 30% of the area adjacent to the perimeter is one region, and the rest divided 50% into an innermost area immediately surrounding the nucleus and a third area in between these. We calculated extensive Cell Painting properties of all 5 fluorescence channels with the SER scale set to 2 px. The results table included these Cell Painting properties for each cell, as well as the object count and all statistics on all properties.

For the GR assay, glucocorticoid receptor translocation was quantified using the following analysis pipeline in Harmony 5.1. It was performed separately for each of the four fields of view in each well. The images underwent a max projection and advanced flatfield correction (to correct for uneven illumination at the corners). The find nuclei module was run on the Hoechst image using method B, with a common threshold of 0.62, predicted area >30 µm^2^, splitting coefficient of 7, an individual threshold of 0.4, and contrast ratio >0.1. The find cytoplasm module was then run using method F, for which we specified the TRITC phalloidin as the membrane channel and the AlexaFluor 488 anti-GR as being in the cytoplasm, with an individual threshold of 0.3. The cells were then filtered to remove the border objects. For each cell, we calculated 1 nuclear Hoechst property (intensity sum), 5 nuclear GR properties (intensity mean, median, sum, 50% quantile, and contrast), and 5 cytoplasmic GR properties (intensity mean, median, sum, 50% quantile, and contrast). We then calculated the percent of the cells that could be considered to have nuclear-enriched GR signal, using the following six variables as selection criteria:

- GR nuc sum/Hoechst nuc sum > 0.35
- GR nuc sum/GR Cyto sum > 0.25
- GR nuc mean/GR Cyto mean > 1.7
- GR nuc median/GR Cyto median > 1.7
- GR nuc quantile/GR Cyto quantile > 1.7
- GR nuc contrast/GR Cyto contrast > 0.3

### HistDiff

The single-cell quantifications were processed into phenotypic fingerprints composed of a vector of normalized values for each well with a {https://github.com/LokeyLab/HistDiff_standalone} refactored version of the HistDiff algorithm^2^. Briefly, a histogram of the extracted cell-by-cell values for each feature in each well is constructed and then smoothed and normalized. Following this, the histograms are compared to the corresponding feature histogram constructed with a defined reference well population, evaluating to the dimensionless HisDiff score which describes how different the feature treatment population(s) compares to the reference population. The HistDiff output data for our screen is available at DOI:10.5281/zenodo.19501979 and described in the first tab of the Source Data file.

In a few plates, there were visibly evident stripe/s through the plate where staining was more intense. Since our many reference controls were randomly located throughout the plate, we could divide the plate into datasets, one for the dim rows and another for the bright rows. Each subdivision was processed with HistDiff independently, then concatenated at the end to represent HistDiff fingerprints for the plate.

### CP activity score

The CP Activity Score (CP score), which reflects the aggregate strength of the phenotypic fingerprint, was calculated for each condition as follows:

1 $${CPscore}=\sqrt{{\sum }_{i=\,{features}\,1}^{n\,{features}}{{x}_{i}}^{2}}$$

x = feature, n = total number of features

### Concatenation

The initial HistDiff-processed phenotypic fingerprints consisted of 5880-feature vectors. The two (1 µM and 10 µM) sets of the original 5880-feature HistDiff fingerprints for each treatment condition (compound with or without PMA) were included such that each compound dose treatment had two representatives. To consider the combined condition for each compound dose, the two (with or without PMA) 5880-feature fingerprints were concatenated, resulting in an 11,670-feature fingerprint.

### Feature reduction methods

Extracting the properties of each cell from five channels and multiple cell regions gives rise to thousands (5930) of features, ranging from signal intensity to morphology and texture. In order to streamline downstream data analysis and reduce the dimensionality of the feature set, we used three approaches.

Here, we describe the feature reduction based on collinearity. Uninformative features were then removed as described in Hight et al.^19^, starting with removal of features with zero standard deviation across all treatments. Then, using the ‘remove multicollinearity’ function from the Pycaret package (version 3.3), all feature pairs with Pearson correlation coefficients greater than a threshold parameter of 0.8 were flagged, thereby reducing redundancy between the features. Each pair member with the highest mean correlation to all other features was removed, resulting in the 482-feature CP fingerprint for each condition (with and without PMA) or 964 features for the concatenated dataset, for each treatment dose (1 µM or 10 µM). These 964-feature CP fingerprints derived from this collinearity reduction approach were used for clustered heatmaps and for quantifying the strength of phenotypic response in the study.

Other approaches to feature reduction were principal component analysis (PCA) and Uniform Manifold Approximation and Projection (UMAP - narrowing it to the final count of 100 projections)^20^. UMAP composite features were generated using the correlation metric and neighbors parameter of 10, with all other parameters left to default, including setting the initial seed to a set integer of 15. Based on the UMAP features, a similarity matrix was created and used for clustering with Hierarchical Density-Based Spatial Clustering of Applications with Noise (HDBSCAN)^20^. See the supplementary methods section of Supplementary Information for more details on feature reduction, UMAP, and HDBSCAN^33^.

### Kolmogorov–Smirnov (K-S) test

For the pilot, we assessed which methods were best at assigning compounds to target classes by performing a two-sample, one-sided Kolmogorov–Smirnov (K-S) test on the colinearity-reduced features from HistDiff. The K-S test compares the significance for in-class versus out-of-class target annotation, with Pearson correlation as the similarity metric, represented as transformed *p*-values. A significant (*p*-value < 0.01) K-S score for a given class indicates that compounds annotated within that class have HistDiff fingerprints that are significantly higher in similarity to each other than to the other phenotypically active compounds in the library under the same condition. This test was used to compare stain sets, cell lines, and activation to optimize the conditions for the screen. We also used the K-S test to assess which feature reduction methods preserved or improved target-class discrimination.

### Quality control ChP4: QCGauntlet

We developed a Python tool for CP quality control, which is available in the {https://github.com/LokeyLab/QCGauntlet} {LokeyLab GitHub repository}. This tool, named QCGauntlet.py, visualizes the CP Activity Scores across the entire screen by generating scatter plots. In these plots, each point represents a single CP Activity Score, with the x-axis corresponding to scores from the PMA condition and the y-axis representing scores from the no PMA condition. Although the tool offers several functions, it was primarily used to display and compare these conditions. To enhance clarity, experimental compounds, and control wells were differentiated using distinct colors and shapes. Two threshold lines—one horizontal and one vertical—were added at the predefined CP Activity Score threshold (see IQR Threshold section), with any score above this threshold classified as active. For more information on ChP4, see Supplementary Fig. S1 and supplementary discussion in Supplementary Information.

The layout of our plate map further informed the expected distribution of controls. For the 1 µM screen, wells with no PMA on the noPMA plate were paired with wells containing PMA on the PMA plate, and vice versa. This design resulted in control wells clustering near the origin, while the JUMP control wells appeared in the top right quadrant. In contrast, the 10 µM screen featured reference control locations (no PMA on the noPMA plate paired with PMA on the PMA plate) expected near the origin and activation control wells (PMA on the noPMA plate paired with no PMA on the PMA plate) expected in the top right corner of the scatter plot.

### Quality control ChP5

In case the control wells were behaving abnormally - having too high CP Activity Score (for reference controls: DMSO vehicle controls with no compound) or too low CP Activity Score (for activation controls: PMA wells in no-PMA plate or no-PMA well in PMA plate) - the following scenario was applied: If more than 10% (3 wells) of the control wells score abnormal, then they are removed, and plate normalization scoring is recalculated, excluding those wells from the control population. If there are still 3 or more (10%) control wells with an abnormal activity level, the whole plate is to be rescreened. If a plate has >22% of all the control wells with an abnormal activity level at the outset, the plate is assigned to be repeated.

### Establishing thresholds of illuminated space

Traditionally, CP Activity Score thresholds were determined using the elbow method. In this approach, CP Activity Scores are sorted in ascending order to create an elbow plot, and the inflection point (large stars in the inset of Fig. 3C) of this curve is identified as the threshold. The phenotypic space covered using this method is visualized on Supplementary Fig. S2B.

Alternatively, we explored a False Discovery Rate (FDR) approach. In this method, we evaluated the CP Activity Scores of the reference controls and excluded the top 5% of controls as outliers. The highest score among the remaining controls was then used as the CP Activity Threshold (small stars in the inset of Fig. 3C). This approach is based on the expectation that controls should theoretically exhibit CP Activity Scores near zero since they are not affected by the activator. Therefore, this method emphasizes compounds with the most pronounced stimulation, potentially overlooking those with more subtle activity. Consequently, any compound demonstrating activity beyond that observed in the reference controls is considered active. The phenotypic space covered using this method is visualized on Supplementary Fig. S2A.

The CP Activity Score threshold was also established using the interquartile range (IQR) method to exclude outlying control values. Outliers were defined as scores above the third quartile (75th percentile), as these did not conform to the general bell-curve distribution of the control data (medium stars in the inset of Fig. 3C). The highest-scoring control within the acceptable IQR range was then used as the threshold value. This approach ensured that only compounds with CP Activity Scores exceeding this threshold were considered phenotypically active. The phenotypic space covered using this method is visualized in Fig. 3B.

### Clustering

We generated clustered heat maps using a multistep process to further explore the phenotypic signatures derived from our cytological profiling data. First, the fingerprint or feature-reduced raw data were exported as tab-delimited files. We then performed hierarchical clustering on both features and compounds using the BioPython treecluster function. Specifically, we employed the complete linkage method in conjunction with the Pearson correlation coefficient as the distance metric to group similar profiles and reveal underlying patterns. The resulting clustered data were visualized in Java TreeView, which facilitated interactive exploration and annotation of the heatmaps. This streamlined approach enabled us to efficiently discern the relationships among compounds and cytological features, complementing our statistical assessments with a clear visual representation of the data.

In addition to heatmaps, we conducted a broader clustering analysis to better understand the target class assignments. First, to determine which feature reduction approach was better for target class assignment, each feature reduction method (colinearity reduction, PCA, and UMAP), we used the K-S test to compare the pairwise similarities of the compound fingerprints within each annotated target class with their similarities to all other compounds outside that class. For each data reduction method, we counted the number of target classes whose significance was at or less than a *p*-value of 0.01. 100 UMAP composite features produced 222 significantly enriched target classes, which performed as well as the 964-feature fingerprints obtained from the collinearity method (219 enriched classes) and much better than PCA (197 enriched classes), even at a threshold of 99% of the variance captured (this threshold for the PCA required 292 composite features). Therefore, for computationally intensive analyses where we needed a small dataset, we used the 100 UMAP composite features, including for our broader clustering by Hierarchical Density-Based Spatial Clustering of Applications with Noise (HDBSCAN) clustering^33^. This was done by generating a similarity matrix of the fingerprints using the Pearson correlation. The resulting similarity matrix was then subjected to HDBSCAN clustering to identify distinct groups within the data. The clusters obtained from HDBSCAN were subsequently visualized using Cytoscape, with an organic layout applied to the graph to enhance the interpretability of the relationships among compounds and cytological features. This integrated approach allowed us to efficiently discern complex patterns in the data, complementing our statistical assessments with clear visual representations.

### Reporting summary

Further information on research design is available in the Nature Portfolio Reporting Summary linked to this article.

## Supplementary information

Supplementary Information

Description of Additional Supplementary Files

Supplementary Dataset 1

Reporting Summary

Transparent Peer Review file

## Source data

Source Data

## Data Availability

All source data is provided as a Source Data file. The list of compounds in the TargetMol library used in the large screen is provided in Supplementary Data 1. The quantified image feature data generated in this study have been deposited in the Zenodo database under https://doi.org/10.5281/zenodo.1950197. Additional layers of processed source data are provided in the Source Data and are also available at https://doi.org/10.5281/zenodo.19500370. The raw image data were too large to be included here (over 10 million.tiff files); access can be obtained by contacting the corresponding authors. Source data are provided with this paper.

## Code availability

The specific version of the code used to perform the analyses and generate results in this study is publicly available and is archived in Zenodo at Zenodo.org under Creative Commons Attribution 4.0 International license. QCGauntlet is under https://doi.org/10.5281/zenodo.19410518, HistDiff is under https://doi.org/10.5281/zenodo.19410007, and all other code is under https://doi.org/10.5281/zenodo.19500370.
