# Supplementary material for: Cell Painting in activated cells illuminates phenotypic dark space and drug mechanisms of action
Source: Nat Commun. 2026 Aug 14;17:9756. doi: 10.1038/s41467-026-76252-6 (PMC13572403; doi:10.1038/s41467-026-76252-6)
Supplement: Supplementary file 1 [file 41467_2026_76252_MOESM1_ESM.pdf]

Supplementary Information for

Cell Painting in activated cells illuminates phenotypic dark space and drug mechanisms of action

Matylda A Zietek, Akshar Lohith, Derfel Terciano, Beverley M Rabbitts, Aswad Khadilkar, John B MacMillan, R Scott Lokey

List of Abbreviations

A549 - a human male lung carcinoma epithelial cell line
AGP - Actin/Golgi/Plasma membrane
BF – Brightfield
ChP - checkpoints for quality control
CP - Cell Painting
CP Activity Score – Cytological Profile Activity Score
DMSO - dimethyl sulfoxide
EGF - epidermal growth factor
ER – endoplasmic reticulum
FDR - false discovery rate
GR - glucocorticoid receptor
HDBSCAN - Hierarchical Density-Based Spatial Clustering of Applications with Noise
HTS - high-throughput screen
iNOS - nitric oxide synthase
IQR - interquartile range
JUMP - Joint Undertaking for Morphological Profiling
K-S test - Kolmogorov–Smirnov test
LPS - lipopolysaccharide
MoA - mechanism of action
PCA – Principal Component Analysis
PKC - protein kinase C
PMA - phorbol myristate acetate
QC – Quality Control
U2OS - a human female tibia bone osteosarcoma epithelial cell line
UMAP - Uniform Manifold Approximation and Projection

Supplementary Figures

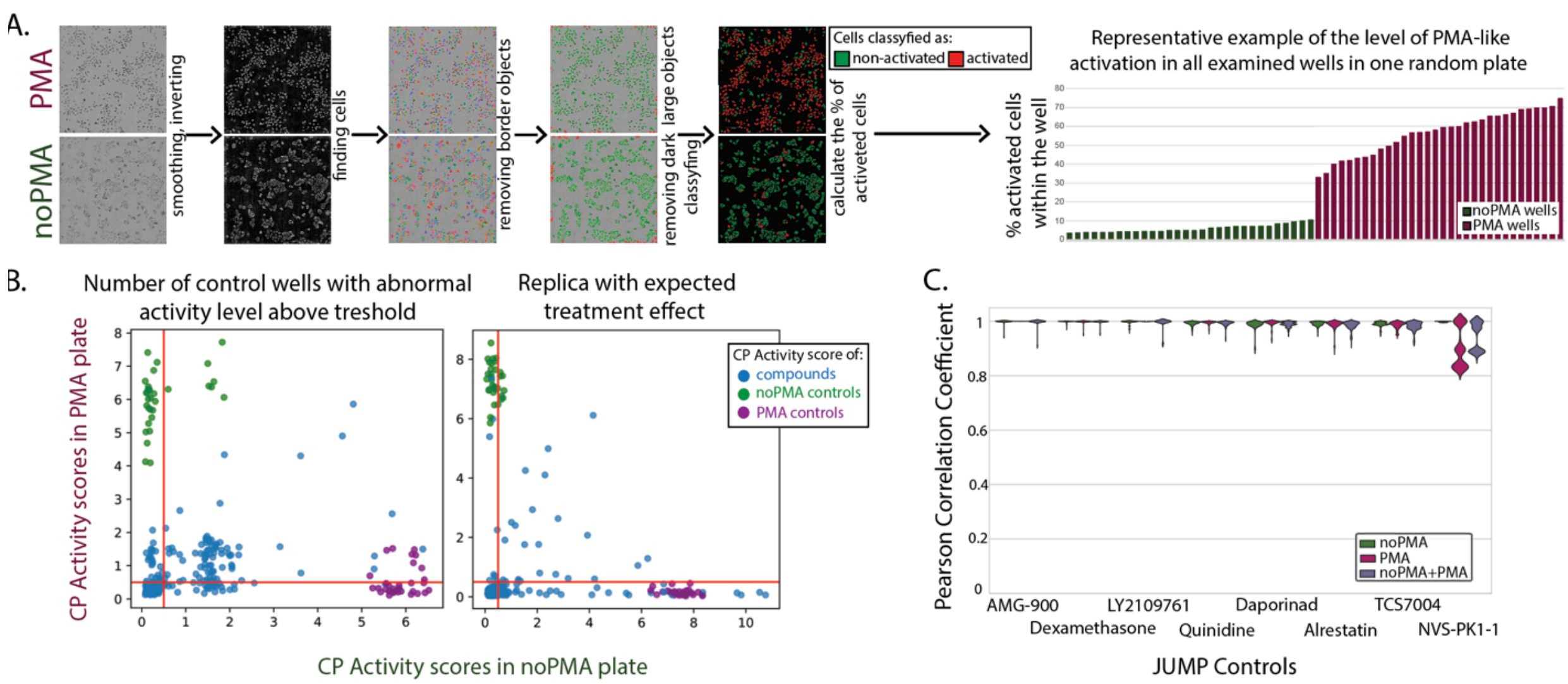

Supplemental Figure S1. Screen quality control. A. For checkpoint 2, representative brightfield images (left) of live cells in resting (green) and activated (maroon) control wells, the linear classifier methods (middle) used to assign resting or activated phenotypes, and data from a representative screen plate (right). **B.** For checkpoint 4, we compared the magnitude of phenotypes (CP Activity Score) within each pair of plates, where one plate had compound-treated resting cells (*x-axis*), and the paired plate had compound-treated PMA-activated cells (*y-axis*). Shown is the QCGauntlet output's scatter plot of wells (dots) for one of the plate pairs that failed the checkpoint (left) and then the replicated experiment that passed the checkpoint (right). **C.** Violin plots of fingerprint similarity across the 10 μM screen (*y-axis*) for 8 positive control compounds (*x-axis*, in descending order of correlation) that were included on every plate of the 10 μM screen in triplicate. Shown are the data when considering only the 27 plates with resting cells (green), only the 27 plates with activated cells (maroon), or the entire 54-plate dataset (periwinkle color).

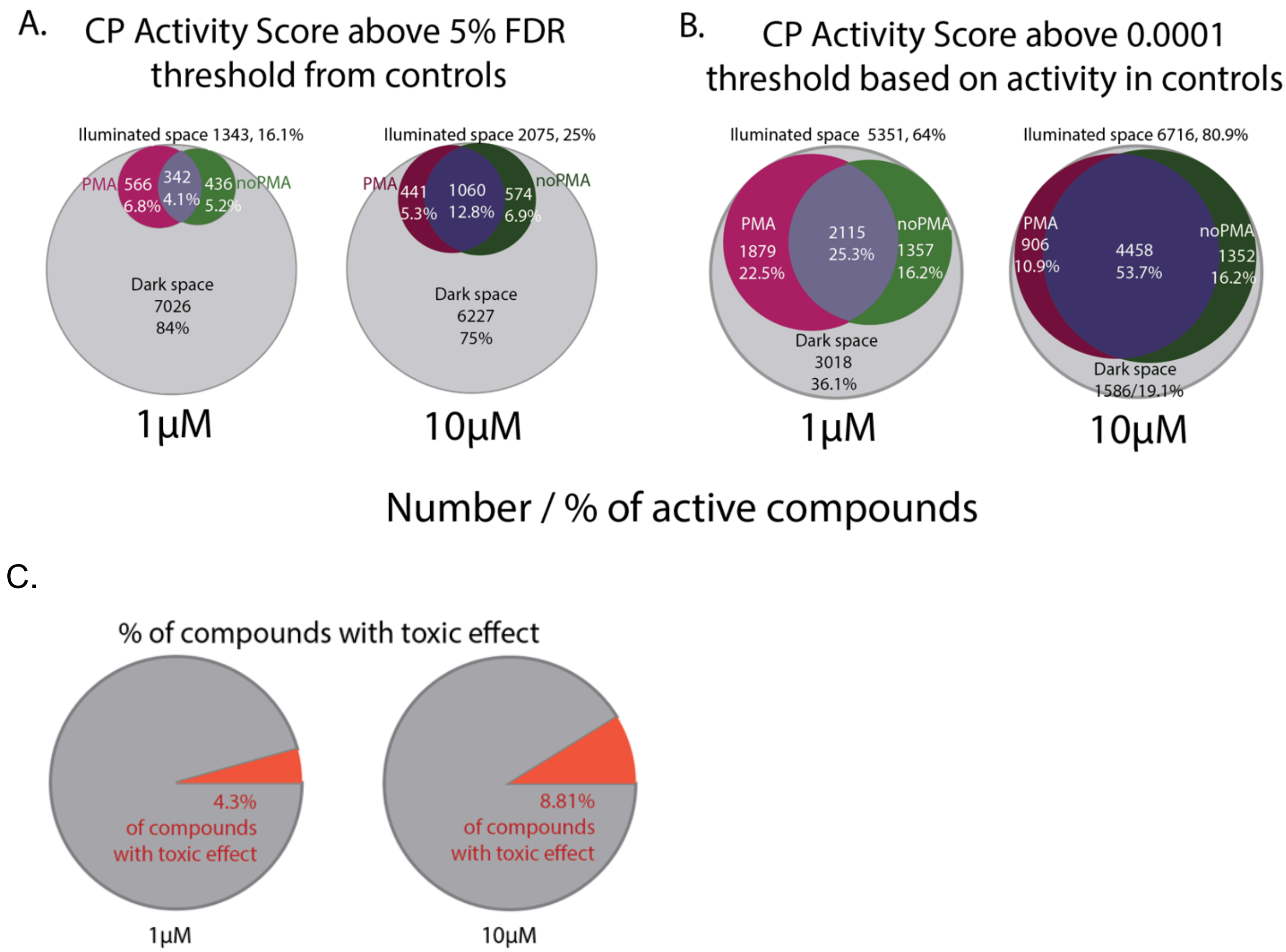

Supplemental Figure S2: Illuminated space analysis for two alternate methods to assign if a compound has a positive phenotype. Venn diagrams as in fig.3B, of all of the compounds tested (largest circle), showing how many, comma, and what percent of compounds had no significant phenotypic differences (grey) compared to no-compound reference controls, a phenotype only in the resting cells (green), a phenotype only in the PMA-activated cells (maroon), or a phenotype in both conditions (violet). **A.** Here, the cutoff for a positive phenotype was the CP Activity Score threshold at which 5% of reference control wells would be called positive (i.e. 5% FDR, small stars in the inset on fig.3C) for 1 μM (left) or 10 μM (right) compound. **B.** Here, the cutoff for a positive phenotype was determined by the elbow method, which identifies the position just above the CP Activity Score inflection point of an elbow plot when CP Activity Scores are sorted in ascending order (large stars in the inset on fig.3C). **C.** Pie charts showing the fraction of compounds with less than half the number of cells of the average control well, for all conditions tested (PMA and no PMA).

Supplementary Tables

Supp. Table S1: HTS Table

Category	Parameter	Description
Assay	Type of assay	Cell-based
	Target	All mammalian targets
	Primary measurement	Image-based detection of cellular features, as stained with fluorescent dyes
	Key reagents	Stains that localize to:<br>● DNA (Hoechst 33342, Life Technologies #H3570)<br>● Mitochondria (MitoTracker Deep Red FM, Invitrogen #M22426)<br>● F-actin (Glu7-PhalloidinTMR,(synthetized in house)<br>● RNA (SYTO14, Fisher #S7576)<br>● ER (Concanavalin A-Alexa Fluor 488 Conjugate, Thermo Fisher Scientific #C11252)<br>● Golgi/plasma membrane (Wheat Germ Agglutinin (WGA)-Alexa Fluor 555 Conjugate, Thermo Fisher Scientific #W32464)
	Assay protocol	Cimini, B. A. et al. Optimizing the Cell Painting assay for image-based profiling. Nature Protocols 18, 1981-2013 (2023).<br><br>Exceptions to the above protocol: we incubated with 1 or 10 uM compound for 24h before fixation, and we included our own set of controls in our own plate map. Imaging was with an Opera Phenix Plus and quantification with Signals Image Artist.
	Additional comments	The entire set of plates were run in two side-by-side sets, one set having no activation and the other set having 50 nM PMA activation for 4h before fixation.
Library	Library size	8,387 compounds
	Library composition	Known bioactives
	Source	TargetMol
	Additional comments	Plated into Echo-compatible Labcyte 384-well plates at a working concentration of 2.5 mM in 90% DMSO, stored at 4 degrees C.
Screen	Format	384-well plates with 320 library compounds per plate and the remaining wells used for controls
	Concentration(s) tested	1 and 10 uM
	Plate controls	● 32 reference controls (DMSO vehicle; these had PMA if on the “PMA plate” where all library compounds were in the presence of PMA, otherwise no PMA)<br>● 6 positive controls for PMA activation (these were treated with PMA if on the “no PMA” plate, or untreatde if on the PMA plate)<br>● 3 replicates each of 8 positive control compounds suggested by the JUMP consortium: AMG 900, NVS-PAK1-1, Alrestatin, Daporinad (fk866, Apo866), TCS 7004 (Tocris Bioscience).

		Desametasone (C22H29FO5), LY2109761, Quinidine<br>• 2 dispensing controls (to verify that Echo dispensing occurred at the two dosing steps).
	Reagent/ compound dispensing system	Bulk dispensing with the BioTek EL406 using a 10 ul peristaltic cassette. Compound dispensing with the Beckman Coulter Echo 650.
	Detection instrument and software	Revvity Opera Phenix Plus with Harmony 5.1 software, quantified with Signals Image Artist software v1.1.
	Assay validation/QC	See our section on quality control in the supplemental information - plates that failed were aborted and repeated. 4 QC checkpoints:<br>1) Inspect by brightfield, after cells adhere and after compound treatment.<br>2) Linear classification of PMA activation from 38 control wells, after PMA addition.<br>3) Whole plate merged montage inspection for edge effects, banding, striping, or other systematic issues.<br>4) Assessed an activity score scatter plot for each pair of plates (with and without PMA) using HistDiff CP Activity Score and QCGauntlet.<br>5) Removal of up to 3 outlier reference control wells, then HistDiff recalculated.
	Correction factors	Histograms of single cell data were compared across treatments using HistDiff, to generated a corrected, normalized histdiff score for each feature.
	Normalization	(see above, HistDiff)
	Additional comments	Screens were performed at UCSC Chemical Screening Center, RRID:SCR_021114.<br><br>The specific version of the code used to perform the analyses in this study is archived in Zenodo: QCGauntlet is under https://doi.org/10.5281/zenodo.19410518, HistDiff is under https://doi.org/10.5281/zenodo.19410007, and all other code is under https://doi.org/10.5281/zenodo.19500370.
Post-HTS analysis	Hit criteria	Results were visualized by heatmaps and types of clustering with different feature reduction strategies, as well as entropy analysis.
	Hit rate	Not applicable.
	Additional assay(s)	2 compounds clustering with glucocorticoid receptor modulators were assessed for ability to modulate glucocorticoid receptor localization.
	Confirmation of hit purity and structure	
	Additional comments	The single-cell image feature data generated in this study have been deposited in the Zenodo database under https://doi.org/10.5281/zenodo.1950197.

Supp. Table S2: SelleckChem sublibrary compounds used in pilot.

Compound	HighScoring Class		Compound	MedScoring Class
**DCC-2036 (Rebastinib)**	ABL		CCT128930	Akt
**GNF-2**	ABL		GDC-0068	Akt
**GNF-5**	ABL		GSK690693	Akt
**GZD824**	ABL		MK-2206 2HCl	Akt
**Imatinib Mesylate (STI571)**	ABL		PF-04691502	Akt
**Ponatinib (AP24534)**	ABL		TIC10	Akt
**Dexmedetomidine**	Adrenergic.Receptor.agonist		ABT-263 (Navitoclax)	BCL
**Epinephrine Bitartrate**	Adrenergic.Receptor.agonist		ABT-737	BCL
**Isoprenaline HCl**	Adrenergic.Receptor.agonist		AT101	BCL
**Mirabegron**	Adrenergic.Receptor.agonist		Gossypol	BCL
**Ritodrine HCl**	Adrenergic.Receptor.agonist		HA14-1	BCL
**Tetrahydrozoline HCl**	Adrenergic.Receptor.agonist		Obatoclax Mesylate (GX15-070)	BCL
**CYC116**	Aurora.Kinase		AGI-5198	Dehydrogenase
**Danusertib (PHA-739358)**	Aurora.Kinase		Azathioprine	Dehydrogenase
**MK-5108 (VX-689)**	Aurora.Kinase		CPI-613	Dehydrogenase
**MK-8745**	Aurora.Kinase		Gimeracil	Dehydrogenase
**MLN8054**	Aurora.Kinase		MK-8245	Dehydrogenase
**PHA-680632**	Aurora.Kinase		Mycophenolate Mofetil	Dehydrogenase
**Lornoxicam**	COX		3-Deazaneplanocin A (DZNeP)	Histone.Methyltransferase
**Mesalamine**	COX		Entacapone	Histone.Methyltransferase
**Naproxen**	COX		EPZ-6438	Histone.Methyltransferase
**Phenacetin**	COX		EPZ5676	Histone.Methyltransferase
**Sulfasalazine**	COX		MM-102	Histone.Methyltransferase
**Triflusal**	COX		SGC 0946	Histone.Methyltransferase
**1-Azakenpaullone**	GSK-3		Asiatic Acid	p38.MAPK
**AR-A014418**	GSK-3		LY2228820	p38.MAPK
**AZD1080**	GSK-3		PH-797804	p38.MAPK
**AZD2858**	GSK-3		SB202190 (FHPI)	p38.MAPK
**BIO**	GSK-3		SB203580	p38.MAPK
**CHIR-99021 (CT99021) HCl**	GSK-3		Skepinone-L	p38.MAPK

**Azacyclonol**	Histamine.Receptor	AG-14361	PARP
**Azatadine dimaleate**	Histamine.Receptor	AZD2461	PARP
**Bepotastine Besilate**	Histamine.Receptor	Iniparib (BSI-201)	PARP
**Betahistine 2HCl**	Histamine.Receptor	INO-1001	PARP
**Brompheniramine hydrogen maleate**	Histamine.Receptor	ME0328	PARP
**Clemastine Fumarate**	Histamine.Receptor	Olaparib (AZD2281; Ku-0059436)	PARP
**17-AAG (Tanespimycin)**	HSP	Ciprofibrate	PPAR
**AT13387**	HSP	GW9662	PPAR
**AUY922 (NVP-AUY922)**	HSP	Rosiglitazone	PPAR
**CH5138303**	HSP	Rosiglitazone HCl	PPAR
**Ganetespib (STA-9090)**	HSP	Rosiglitazone maleate	PPAR
**HSP990 (NVP-HSP990)**	HSP	T0070907	PPAR
**CYT997 (Lexibulin)**	Microtubule.Associated	Clopamide	Sodium.Channel
**Docetaxel**	Microtubule.Associated	Dibucaine HCl	Sodium.Channel
**Epothilone A**	Microtubule.Associated	Ibutilide Fumarate	Sodium.Channel
**Epothilone B (EPO906; Patupilone)**	Microtubule.Associated	Oxcarbazepine	Sodium.Channel
**Nocodazole**	Microtubule.Associated	Phenytoin sodium	Sodium.Channel
**Paclitaxel**	Microtubule.Associated	Procaine HCl	Sodium.Channel
**Cilazapril Monohydrate**	RAAS	FH535	Wnt.beta-catenin
**Clinofibrate**	RAAS	IWP-2	Wnt.beta-catenin
**Enalapril Maleate**	RAAS	IWP-L6	Wnt.beta-catenin
**Moexipril HCl**	RAAS		
**Olmesartan Medoxomil**	RAAS		
**Quinapril HCl**	RAAS		
**Ki8751**	VEGFR.KIT.PDGFR		
**KRN 633**	VEGFR.KIT.PDGFR		
**Pazopanib**	VEGFR.KIT.PDGFR		
**Regorafenib (BAY 73-4506)**	VEGFR.KIT.PDGFR		
**Telatinib**	VEGFR.KIT.PDGFR		
**Tivozanib (AV-951)**	VEGFR.KIT.PDGFR		

Supp. Table S3: Positive control compounds in 10μM screen

Name	Supplier	Cat #
**AMG 900**	Cayman Chemical Company	19176-1
**NVS-PAK1-1**	Cayman Chemical Company	19964-5
**Alrestatin**	Cayman Chemical Company	29888-5
**Daporinad (fk866, Apo866)**	Fisher	501362977
**TCS 7004, Tocris Bioscience**	Fisher	508810RD
**Desametasone, C22H29FO5**	Fisher	MP21993506
**LY2109761**	Sigma	SML2051-5MG
**Quinidine**	Spectrum	TCI-Q0006-5G

Supp. Table S4: List of all stains used

Name	Supplier	Cat #	Clone or Lot
**Hoechst 33342**	Life Technologies	H3570	
**MitoTracker Deep Red FM**	Invitrogen	M22426	
**Glu7-PhalloidinTMR**	(synthetized in house)		
**EdU**			
**iNOS (D6B6S) Rabbit mAb**	Cell Signaling Technologies	13120S	Clone D6B6S-13120S, lot 7
**GM130 mouse**	Fisher	BDB610823	Lot 9322749 or 8033959
**alpha-tubulin-FITC antibody mouse monoclonal**	Sigma	F2168	Clone DM1A, batch 88052
**Phospho histone H3 (Ser10) Recombinant Rabbit Monoclonal Antibody (9H12L10)**	Invitrogen	701258	Clone 9H12L10, lot 2055236 or RK245775
**Chicken anti-Mouse IgG (H+L) Cross-Adsorbed Secondary Antibody Alexa Fluor 488**	Life Technologies	A21200	Lot 2140649
**Chicken anti-Rabbit IgG (H+L) Cross-Adsorbed Secondary Antibody Alexa Fluor 647**	Life Technologies	A21443	Lot 2306791
**SYTO14**	Fisher	S7576	
**Concanavalin A, Alexa Fluor 488 Conjugate**	Thermo Fisher Scientific	C11252	
**Wheat Germ Agglutinin (WGA) Alexa Fluor 555 Conjugate**	Thermo Fisher Scientific	W32464	
**Mouse anti-GR G-5, RRID:AB_2687823**	Santa Cruz Biotech	sc-393232	Clone G-5

Supp. Table S5: Stepwise staining methods

	CP Screen and Pilot	GR assay	Pilot (“classic” stains)	
	(JUMP stains)		“cyto” stain set 1	“EdU” stain set 2
**Live stain(s)**	500 nM MitoTracker Deep Red FM in complete media, 30 min 37°C 5% CO_2	Not done		20 µM EdU and 100 nM MitoTracker Deep Red FM in complete media, 1 h 37°C 5% CO_2
**Fix**	4% formaldehyde, 6 min RT	4% formaldehyde, 20 min RT		
**Wash**	Twice in PBS			
**Permeabilize and block**	Not done	0.1% Triton X-100, 1% BSA, 1 h RT	0.5% Triton X-100, 2% bovine serum albumin in PBS, 0.5 h RT dark	
**Wash**	Not done	Twice in cold PBS	Twice in PBS	
**Click reaction**	Not done			1 mg/mL TMR-azide, 4 mM $CuSO_4$, 2 mg/mL sodium ascorbate in 100 mM Tris
**Wash**	Not done			Twice in PBS
**Primary stain**	8.25 nM TMR-Phalloidin, 5 µg/ml Concanavalin A, 1 µg/ml Hoechst, 1.5 µg/ml WGA, 6 µM Syto14 in 0.1% Triton X-100, 1% BSA 17 min RT dark	mouse anti-GR (or no primary controls) in BSA/PBS o/n 4 ℃	1:5,000 rabbit anti-histone H3 phospho-S10, 1:1,000 mouse anti-GM-130, 2% BSA/PBS o/n 4 ℃	1:5,000 mouse anti-tubulin α, 2% BSA/PBS o/n 4 ℃
**Wash**	Twice in PBS			
**Secondary stain**	Not done	2 µM Hoechst, 7 nM TMR-phalloidin, 1:1,000 AF647-anti-rabbit, 1:1,000 AF488-anti-mouse, 2 h RT dark		2 µM Hoechst in 2% BSA/PBS 2 h RT dark
**Wash**	Not done	Twice in cold PBS	Twice in PBS	
**Mounting medium**	0.05% Na Az in PBS		0.1% Na Az in PBS	

Supp. Table S6: Opera Phenix imaging setup

	Excitation laser and emission filter wavelengths (nm)	Pilot with classic "cyto" stain set 1	Pilot with classic "EdU" stain set 2	Pilot with JUMP stain set and CP screen	GR assay
**B1**	405, 435-480	95% 120 ms	95% 100 ms	n/a	95% 100 ms
**B2**	405, 435-550	n/a	n/a	95% 160 ms	n/a
**G1**	488, 500-530	n/a	n/a	95% 160 ms	n/a
**G2**	488, 500-550	95% 500 ms	95% 200 ms	n/a	50% 100 ms
**Y**	488, 515-550	n/a	n/a	95% 60 ms	n/a
**R**	561, 570-630	95% 80 ms	95% 20 ms	95% 160 ms	95% 80 ms
**M**	640, 650-760	95% 400 ms	95% 100 ms	95% 40 ms (pilot) or 20 ms (screen)	95% 400 ms
**BF**	Brightfield 650-760 nm	95% 40 ms	50%, 100 ms	95% 40 ms	50% 100 ms
**Sequence of capture (camera1/camera2)**		B1/G2, R/M, BF		G1, Y, R/M, B1/BF	B1/G2, R/M, BF

Supplementary Discussion

A robust Quality Control method increases confidence in HTS CP datasets and decreases screening cost.

Generating high-quality datasets from CP HTS requires several QC checkpoints (*ChP*) along the screen pipeline.[4] Quickly detecting artifacts prevents working with faulty data, lowering cost and labor. An initial checkpoint (*ChP1* Fig.2A) was developed to monitor cell health using live-cell (brightfield) imaging of a few randomly picked plates for each batch, conducted before compound treatment and again before PMA activation.

Next, in a second checkpoint (*ChP2*), before fixation, all 38 control wells per plate (32 negative and 6 positive, Fig.2A, red and blue squares, respectively, in the 'Compound dosing to cell culture' panel section) were imaged in brightfield and processed using a machine learning-based linear classification step (Supp.Fig.S1A). This allowed detection of the level of activation in every plate/well examined and identification of assay plates that had behaved abnormally (showing low activation after PMA treatment or high activation in the presence of vehicle only), thereby allowing such assay plates to be excluded from subsequent steps in the workflow.

A representative plate is shown in the bar graph in Supp.Fig.S1A to the right, where it is shown that wells without activator (noPMA, green bars) have less than 10% of cells

behaving like activated ones, whereas most of the wells activated with PMA (maroon bars) have more than 50% of such cells.

The next QC checkpoint (*ChP3*) was conducted on the final multichannel, Z-stack CP images. We made an image montage of each 384-well plate and visually examined them to detect any plate artifacts. In case of serious problems, the dataset was discarded and repeated from the cell plating onwards. In a few cases where sections of the plate have obvious technical artifacts like banding or striping, data could be recovered by block normalization, where the large number of reference wells (32) allows for sufficient representation after subdividing the plate, treating the subdivided sections independently.

We then used fingerprints from a single-cell based algorithm (HistDiff)[2] to calculate the CP Activity Score, which represents how different each well is compared to the set of reference control wells. This score was used in a QC checkpoint (*ChP4*) for which we developed an application QCGauntlet.py (https://github.com/LokeyLab/QCGauntlet) for visualization facilitating the assessment of overall response to compounds and PMA activator within a plate. QCGauntlet generates various visualizations for CP Activity Scores, with one primary use-case being the generation of scatter plots to compare CP Activity Scores between two different conditions, particularly PMA-treated versus untreated plates (Supp.Fig.S1B). This analysis was done for all 54 pairs of plates.

Additionally, the app provides a comprehensive overview of the entire experiment by visualizing CP Activity Scores across all plates. For each compound dose (1 and 10 μM), our screen had 27 plate pairs (with and without PMA), and the tool allowed us to view the CP Activity Scores of all these plates in a single, consolidated figure consisting of scatter plots for each individual plate, allowing identification of potential batch effects or plate anomalies, thus ensuring data integrity and reliability.

An example of a plate rejected at this checkpoint is shown in Supp.Fig.S1B, where > 22% of all control wells have abnormal activity levels; this plate was assigned to be repeated. On the right is a scatter plot for replica showing the expected overall response to treatment: some compounds show distinguished phenotype just in noPMA condition (higher values on the X-axis), some in just PMA (higher values on the Y-axis), some in both (around diagonal of the plot). There is also dark space represented by compounds in the left-bottom corner of the scatter.

Unfortunately, due to the cost and complexity of CP and related approaches, replicates of the whole screen were not feasible within the scope of this study. However, we did assess reproducibility by including, in triplicate in random positions on every plate of the 10 μM screen, 8 maximally diverse positive control compounds (Fig.2A, green squares in the ‘Compound dosing to cell culture’ step). We selected a set of compounds (Supp. Table 2) modified from those proposed by the JUMP Consortium (https://github.com/jump-cellpainting/JUMP-Target?tab=readme-ov-file#positive-control-compounds {JUMP Consortium GitHub page}).

The similarity of cell response to these compounds was high across replicas within the plate, between plates, and between batches no matter the condition (PMA *vs.* noPMA) (Supp.Fig.S1C). Such high reproducibility strengthens our confidence in the overall quality of the screen.

Supplementary Methods

Data Preprocessing and Filtering

The analysis began with preprocessing of UMAP (Uniform Manifold Approximation and Projection) data from high-content screening of compounds under different treatment conditions (PMA and noPMA) at two concentrations (1 μM and 10 μM). The analysis pipeline consisted of the following steps:

1. Initial Data Selection: The umap_filtering.py script filtered UMAP data from original high-dimensional phenotypic profiles. Compounds were matched with their target annotations from key files containing threshold CP scores above 1e-4, ensuring only compounds with significant activity were included. The data was organized into four primary datasets: PMA 1 μM, PMA 10 μM, noPMA 1 μM, and noPMA 10 μM.

2. Target Filtering: The filter_targets.py script further refined the datasets by keeping only targets represented by at least 3 compounds, excluding the generic "Others" category. This ensured statistical robustness in subsequent analyses by focusing on well-represented targets. The filtering resulted in distinct sets of compounds for each condition, with detailed target distribution statistics saved for reference.

Entropy Calculation

The entropy analysis was performed using the target_entropy_analysis.py script through the following steps:

1. UMAP-Based Clustering: The script loaded filtered UMAP data for each condition and concentration. For each dataset, two clustering methods were applied:
    - K-Means clustering with 50 clusters
    - HDBSCAN (Hierarchical Density-Based Spatial Clustering of Applications with Noise) with a minimum cluster size of 10

2. Target Entropy Calculation: For each target across the datasets, Shannon entropy was calculated to quantify the spread of compounds belonging to that target across different clusters. Lower entropy values indicate higher consolidation (compounds are concentrated in fewer clusters), while higher entropy values indicate dispersal (compounds are spread across many clusters). Only targets with at least 5 compounds were included in this analysis to ensure statistical reliability.

The Shannon entropy for each target's distribution across clusters was calculated using the following equation:

$$H(T) =- \sum_{i=1}^{N} p_i(p_i) \quad (2)$$

Where:

- · $H(T)$ is the entropy value for target T
- · p_i is the probability of finding a compound with target T in cluster i
- · N is the total number of clusters where the target appears
- · log_2 indicates the base-2 logarithm, so entropy is measured in bits

In the implementation, the probabilities were calculated by:

$$p_i = \frac{n_{i,T}}{\sum_{j=1}^{N} n_{j,T}} \quad (3)$$

Where:

- · $n_{i,T}$ is the number of compounds with target T in cluster i
- · The denominator is the total number of compounds with target T across all clusters

3. Entropy Comparison: The script generated entropy comparison plots and data files (entropy_comparison_1uM.csv and entropy_comparison_10uM.csv), which captured the differences in entropy values between PMA and noPMA conditions for each target at each concentration. A negative entropy difference (PMA - noPMA) indicated greater consolidation of a target in the PMA condition, while a positive difference indicated greater consolidation in the noPMA condition.

The comparison between PMA and noPMA conditions was quantified as an entropy difference:

$$\Delta H = H_{PMA}(T) - H_{noPMA}(T) \quad (4)$$

Where:

- · ΔH is the entropy difference (stored in the 'Entropy_Diff' column of the CSV files)
- · $H_{PMA}(T)$ is the entropy of target T in the PMA condition
- · $H_{noPMA}(T)$ is the entropy of target T in the noPMA condition

A negative value of ΔH indicates that a target is more consolidated (lower entropy) in the PMA condition compared to noPMA, while a positive value indicates the target is more consolidated in the noPMA condition. The implementation used the entropy function from SciPy's scipy.stats module, which computes Shannon entropy according to this formula. In the code, this was applied to the normalized distribution of compounds across clusters for each target, with a minimum threshold of 5 compounds per target to ensure statistical reliability.

Pathway Enrichment Analysis

The pathway enrichment analysis was conducted using the analyze_pathway_enrichment.py script:

1. Target Categorization: Targets were categorized into specific pathway groups based on biological function using a comprehensive classification system. The classification covered major categories including:
   - Neurotransmitter systems (Serotonergic, Dopaminergic, etc.)
   - Ion channels and transporters
   - Kinase subfamilies
   - Nuclear receptor subfamilies
   - GPCRs (G-protein coupled receptors)
   - Various enzyme types
   - Metabolic pathways
   - Cytoskeleton and cellular processes
   - Cell death and survival mechanisms
   - Immune and inflammatory systems
   - Epigenetic regulation
   - Redox systems

2. Pathway Distribution Analysis: The script analyzed the distribution of pathway categories among targets showing significant entropy differences between conditions. It identified pathways that were preferentially consolidated in either PMA or noPMA conditions.

3. Enrichment Calculation: Enrichment scores were calculated as the ratio of the percentage representation of each pathway category in PMA-consolidated targets versus noPMA-consolidated targets. This quantified which biological systems showed the strongest condition-dependent consolidation effects.

Network Visualization

The visualize_cluster_networks.py script was used to create network visualizations that further illustrated the relationships between clusters and targets:

1. Network Construction: Clusters were represented as nodes in a network, with edges connecting clusters based on their proximity in UMAP space. Each cluster was colored based on its most enriched targets, with size proportional to target enrichment.

2. Target Distribution Analysis: For each condition and concentration, target distribution across clusters was analyzed and visualized as heatmaps, providing another perspective on target consolidation patterns.

3. Integrated Visualization: Comprehensive visualizations were created combining UMAP scatter plots, zoomed views of specific clusters, and network representations to highlight the most interesting patterns of target consolidation.

The analysis revealed significant differences in target consolidation patterns between PMA and noPMA conditions, with specific biological pathways showing preferential consolidation in each condition. These findings provided insights into how pathway activation patterns differ under PMA treatment, which activates protein kinase C signaling pathways, and contributes to our understanding of biological system organization in response to different cellular states.
