# Supplementary material for: Cell Painting in activated cells illuminates phenotypic dark space and drug mechanisms of action
Source: Nat Commun. 2026 Aug 14;17:9756. doi: 10.1038/s41467-026-76252-6 (PMC13572403; doi:10.1038/s41467-026-76252-6)
Supplement: Supplementary file 2 [file 41467_2026_76252_MOESM2_ESM.pdf]

<u>Description of Additional Supplementary Files</u>

Supplementary Data 1: List of TargetMol Library Compounds Used In Screen: the well locations, target, and other detailed information for all compounds in our screen, which were purchased as a single library from TargetMol.
