# Supplementary material for: Cell Painting in activated cells illuminates phenotypic dark space and drug mechanisms of action
Source: Nat Commun. 2026 Aug 14;17:9756. doi: 10.1038/s41467-026-76252-6 (PMC13572403; doi:10.1038/s41467-026-76252-6)
Supplement: Supplementary file 3 [file 41467_2026_76252_MOESM4_ESM.pdf]

Corresponding author(s): R. Scott Lokey

Last updated by author(s): May 22, 2026

Reporting Summary

Nature Portfolio wishes to improve the reproducibility of the work that we publish. This form provides structure for consistency and transparency in reporting. For further information on Nature Portfolio policies, see our Editorial Policies and the Editorial Policy Checklist.

Statistics

For all statistical analyses, confirm that the following items are present in the figure legend, table legend, main text, or Methods section.

n/a	Confirmed	
☐	☒	The exact sample size (*n*) for each experimental group/condition, given as a discrete number and unit of measurement
☐	☒	A statement on whether measurements were taken from distinct samples or whether the same sample was measured repeatedly
☐	☒	The statistical test(s) used AND whether they are one- or two-sided<br>*Only common tests should be described solely by name; describe more complex techniques in the Methods section.*
☐	☒	A description of all covariates tested
☒	☐	A description of any assumptions or corrections, such as tests of normality and adjustment for multiple comparisons
☐	☒	A full description of the statistical parameters including central tendency (e.g. means) or other basic estimates (e.g. regression coefficient) AND variation (e.g. standard deviation) or associated estimates of uncertainty (e.g. confidence intervals)
☐	☒	For null hypothesis testing, the test statistic (e.g. *F*, *t*, *r*) with confidence intervals, effect sizes, degrees of freedom and *P* value noted<br>*Give P values as exact values whenever suitable.*
☒	☐	For Bayesian analysis, information on the choice of priors and Markov chain Monte Carlo settings
☒	☐	For hierarchical and complex designs, identification of the appropriate level for tests and full reporting of outcomes
☒	☐	Estimates of effect sizes (e.g. Cohen's *d*, Pearson's *r*), indicating how they were calculated

Our web collection on statistics for biologists contains articles on many of the points above.

Software and code

Policy information about availability of computer code

Data collection	Image data were collected on a Revvity Opera Phenix microscope and preprocessed using the vendor's Harmony and Signals ImageArtist software.
Data analysis	All scripts developed for downstream data analysis are available in the Dryad database.

For manuscripts utilizing custom algorithms or software that are central to the research but not yet described in published literature, software must be made available to editors and reviewers. We strongly encourage code deposition in a community repository (e.g. GitHub). See the Nature Portfolio guidelines for submitting code & software for further information.

Data

Policy information about availability of data

All manuscripts must include a data availability statement. This statement should provide the following information, where applicable:

- Accession codes, unique identifiers, or web links for publicly available datasets
- A description of any restrictions on data availability
- For clinical datasets or third party data, please ensure that the statement adheres to our policy

Links to all source data, deposited in the Zenodo database, are provided in the Excel file Supplemental_Data_Source_Table_Nature.xlsx.

April 2023

Research involving human participants, their data, or biological material

Policy information about studies with human participants or human data. See also policy information about sex, gender (identity/presentation), and sexual orientation and race, ethnicity and racism.

Reporting on sex and gender	n/a
Reporting on race, ethnicity, or other socially relevant groupings	n/a
Population characteristics	n/a
Recruitment	n/a
Ethics oversight	n/a

Note that full information on the approval of the study protocol must also be provided in the manuscript.

Field-specific reporting

Please select the one below that is the best fit for your research. If you are not sure, read the appropriate sections before making your selection.

[x] Life sciences [ ] Behavioural & social sciences [ ] Ecological, evolutionary & environmental sciences

For a reference copy of the document with all sections, see nature.com/documents/nr-reporting-summary-flat.pdf

Life sciences study design

All studies must disclose on these points even when the disclosure is negative.

Sample size	Unfortunately, due to the cost and complexity of CP and related approaches, replicates of the whole screen were not feasible within the scope of this study.
Data exclusions	We describe 4 QC checkpoints in detail in the supplementary information (also in Figure 2 and Supplemental figure S1). Plates were excluded if they had stain striping, lack of PMA response, or inappropriate phenotypes in the control wells (see QC Gauntlet).
Replication	We had one biological replicate of each condition in our screen, but our extensive QC framework, replicated controls, orthogonal validation, and biological coherence provide strong evidence that our dataset captures genuine biological responses.
Randomization	Each plate contained 38 vehicle-only control wells (including a combination of DMSO-only and PMA-only wells in both the PMA-treated and untreated plates) in randomized positions (Fig.2A), providing extensive within-plate references for normalization and quality assessment. All controls were distributed within each plate using a mixed integer linear program (MILP) designed specifically to minimize positional and batch effects.
Blinding	Quantification of the images was performed without any knowledge of compound identities. The TargetMol library that was screened was used in its entirety and was not pre-selected based on any preconceived biological hypothesis.

Reporting for specific materials, systems and methods

We require information from authors about some types of materials, experimental systems and methods used in many studies. Here, indicate whether each material, system or method listed is relevant to your study. If you are not sure if a list item applies to your research, read the appropriate section before selecting a response.

Materials & experimental systems

n/a	Involved in the study	
[ ]	[x]	Antibodies
[ ]	[x]	Eukaryotic cell lines
[x]	[ ]	Palaeontology and archaeology
[x]	[ ]	Animals and other organisms
[x]	[ ]	Clinical data
[x]	[ ]	Dual use research of concern
[x]	[ ]	Plants

Methods

n/a	Involved in the study	
[x]	[ ]	ChIP-seq
[x]	[ ]	Flow cytometry
[x]	[ ]	MRI-based neuroimaging

Antibodies

Antibodies used	All antibodies used in this study are listed in Supplemental Table 3.
Validation	Antibodies were validated by the manufacturer.

Eukaryotic cell lines

Policy information about cell lines and Sex and Gender in Research

Cell line source(s)	A549 and U2OS cell lines were obtained from the UT Southwestern Medical Center cell line repository.
Authentication	A549 and U2OS cell lines were authenticated by the UT Southwestern Medical Center.
Mycoplasma contamination	A549 and U2OS cell lines tested negative for mycoplasma contamination.
Commonly misidentified lines (See ICLAC register)	There are no commonly misidentified cell lines used in this study.

Plants

Seed stocks	n/a
Novel plant genotypes	n/a
Authentication	n/a
