# Supplementary material for: Cell Painting in activated cells illuminates phenotypic dark space and drug mechanisms of action
Source: Nat Commun. 2026 Aug 14;17:9756. doi: 10.1038/s41467-026-76252-6 (PMC13572403; doi:10.1038/s41467-026-76252-6)
Supplement: Supplementary file 4 [file 41467_2026_76252_MOESM5_ESM.pdf]

nature portfolio

Peer Review File

Cell Painting in activated cells illuminates phenotypic dark space and drug mechanisms of action.

Corresponding Author: Professor Robert Lokey

This file contains all reviewer reports in order by version, followed by all author rebuttals in order by version.

Version 0:

Reviewer comments:

Reviewer #1

(Remarks to the Author)
The manuscript "Cell painting in activated cells illuminates phenotypic dark space and uncovers novel drug mechanisms of action" by Zietek et al., performs the now well established Cell Painting assay to screen a collection of bioactive small molecule compounds in the A549 lung cancer cell line under standard cell culture conditions and also conditions which "activate" cells following phorbol myristate acetate (PMA) treatment. The article is well written and includes some nice methodology for quality control and assessment of a Cell Painting activity score. The major conclusions is that cell activation upon PMA illuminates new phenotypic "dark space" following compound treatment that would otherwise be missed under standard cell culture conditions.

General comments: While this is a well conducted study that make the important point that perturbation of cell culture systems influences how cells respond to drug treatment, and in turn will influence the ability of the Cell Painting assay to identify and cluster bioactive compounds by phenotypic profiling -this observation however is not unexpected. Also, the magnitude to which PMA treatment reduces phenotypic dark space appears somewhat limited when using robust activity thresholds or 5% false discovery rate and while performance of drug mechanism-of-action clustering is improved upon PMA stimulation for some therapeutic target classes, it is reduced for other target classes. These results indicate that the approaches described and PMA stimulation specifically may only have a subtle effect upon illuminating phenotypic dark space and while this will likely uncover novel mechanisms-of-action other important mechanisms-of-action may be lost.

Specific points:
The authors state in the discussion that they "demonstrate that activating cells with PMA prior to CP analysis substantially expands the observable phenotypic space and enables the detection of otherwise inactive compounds", however the magnitude of phenotypic space expansion appears somewhat limited. As the authors present in Figure 1C : dark phenotypic space in combined standard and PMA (1micromolar) stimulated conditions is still extremely high at 72%, while with PMA treatment alone dark phenotypic space is 80% compared to 85% in standard cell culture conditions. These results would appear to contradict the authors claim that PMA activation "substantially" expands observable phenotypic space. While the authors show in supplementary Figure 1B that observable phenotypic space can be significantly increased with setting a lower CP activity threshold, as the authors acknowledge, this comes at the cost of increased false positive activity and when using a 5% false discovery rate (supplementary figure 1B) the dark phenotypic space remains even higher at: 84% in combined standard and PMA (1micromolar) stimulated conditions; 93.2% in PMA treatment alone compared to 90.7% in standard cell culture conditions.

Line 254: "The resulting phenotypic fingerprints consist of hundreds of cell features extracted from the segmented regions of the images for each cell and further processed using the HistDiff algorithm..". Can the author clarify here how many features were extracted per cell and whether feature reduction methods has any impact on illuminating novel dark phenotypes in PMA activated cells? E.g. would more features reduce the dark phenotypic space?
Line 309: "Indeed, thanks to PMA efficacy, we can clearly observe the phenotypic effect of PKC, MEK, and ERK regulators in PMA-treated cells, among others, giving us confidence in the screen results (Fig 4D)". I agree the phenotypic effect of these compounds are enhanced in PMA-treated cells which are expected and encouraging with regards to the screening set up. Are the activity of other unexpected target classes enhanced or diminished in PMA stimulated conditions which may provide some novel insights into particular compound classes?

Line 378 “In contrast, other target classes became less consolidated under PMA treatment (Fig.4F, red), reflecting greater phenotypic diversity upon PKC activation.” Could this phenotypic diversity reflect a lack of activity/potency resulting from loss of the primary mechanisms-of-action of compounds that are present in the standard culture conditions, thus now amplifying more subtle off-target compound activities? If this is the case this could confound the utility of some cell activating conditions to effectively identify and predict important drug mechanisms of action from Cell Painting profiles.

Reviewer #2

(Remarks to the Author)
The hypothesis that certain phenotypic responses to compound exposure are only observable under particular cell states is interesting, and worth investigating. However, I have concerns about the overall experimental design, particularly the apparent lack of replicates, and more information on the experimental design is needed to properly evaluate whether the conclusions derived from analysis are sound. The paper structure could also be re-worked to improve readability by focusing more on the main results, and moving details on the pilot / qa/qc to the methods and supplementary.

Large comments:
- Concerned about the lack of replicates. There are not enough experimental design details provided to evaluate how big of an issue this is. There are substantial plate and well position effects in Cell Painting screening studies. Without replicates that are properly randomized across well positions and plates, it is difficult to determine whether an effect is statistically significant and whether it is a true biological effect or confounded by technical factors. Were activated and non-activated samples on different plates? Were the well positions of the same compound the same under activation versus non-activation conditions? Since well position and plate effects are ubiquitous, knowing these details are important for evaluating the downstream data analysis.

- 3 of 5 figures describe the experiment (pilot, overview, qa/qc) while only two focus on the main result of the paper. The amount of text is also proportional here. Suggest greatly condensing the first 60% of the paper (ie. one figure, proportional amount of text), enabled by ample use of the supplementary materials, to put more focus on the main results.

- Many methodological details are repeated across the methods and results section. The paper would be more readable if this was reduced, for example L353-359 can be included in only the methods and replaced with a short statement describing what the main finding of Figure 5A is and why it matters, rather than how it was produced.

Small comments:
- Figure legends are much too long and contain a mix of methods, results, and interpretation

- L354-359: Don’t understand the dimension reduction to UMAP components prior to similarity matrix calculation. UMAP has issues and should be used with many grains of salt; why not use a more reliable method like PCA or just use raw features if the next step is to compute profile:profile similarity and not to directly visualize in low dimensions?

- The “JUMP stain set” is the Cell Painting assay, which refers to the particular set of stains outlined in Bray et al and Cimini et al (both Nat Prot). Suggest calling it the Cell Painting stains to avoid confusion (examples: L120, L129, L137)

Version 1:

Reviewer comments:

Reviewer #1

(Remarks to the Author)
I thank the authors for their responses and clarifications to the original reviewers comments and the efforts they made to revising the manuscript. I restrict my comments and response to the following major outstanding points.

In response to the original comments that the results are not unexpected the authors responded:
"We agree that it is not unexpected that a baseline drug treatment (i.e., PMA) will influence the ability to cluster bioactive compounds by phenotypic profiling; however, our study is the first to evaluate this effect in detail, quantify the results, and evaluate the effect in different cell lines and activators (both PMA and EGF)". I do not disagree with the authors that they are the first to evaluate the effect in detail, however I remain of the opinion that this work does not represent a significant enough novel conceptual advance to merit publication in Nature Communications.

In response to the original comment: The magnitude to which PMA treatment reduces phenotypic dark space appears somewhat limited.... The author responded:
"We think it is more important to consider the combined number of compounds that were phenotypically active under both PMA-stimulated and unstimulated conditions, and to compare that with the results from the unstimulated (classical CP) condition alone. At the 5% false discovery rate (FDR) threshold, combining profiles from resting and PMA-activated cells led to 1,343 active compounds at 1 µM, compared to 778 under the standard (resting-only) condition, a ~1.7-fold increase representing 565 compounds whose phenotypic activity was detectable only upon cell activation. This conservative estimate

increases substantially at less stringent activity thresholds". While the 1.7 fold increase in phenotypic activity of compounds detected upon cell activation is encouraging total dark phenotypic space in combined standard and PMA stimulated conditions is still 72% compared with unstimulated dark phenotypic space at 85%. Reducing stringency threshold and increasing concentration of drugs to 10uM will certainly result in less phenotypic dark space but at the cost of high error rates, multiple off-target activities at non physiologically relevant concentrations. Thus the results while positive still seem somewhat incremental in reducing phenotypic dark space particularly if the methods would be applied in practice at earlier stages of drug discovery where compound potency is typically low.

I agree that the methodology and the results themselves are interesting and worth reporting but i think this work would be better suited to a specialized chemical biology or drug screening journal.

Reviewer #2

(Remarks to the Author)
The paper is much improved and I appreciate the additional details about plate and well position layout - I am reassured that the experimental design does not confound compound effects with these technical factors. I am still skeptical about the single replicate for each condition, but appreciate that the large number of profiled compounds still enables detection of large-scale patterns.

I have a few additional comments:

Cell Painting should be capitalised in the title and key words

I appreciate that the authors moved much of the QA/QC discussion to the supplementary materials, however I think that the paper would be even more compelling and readable if the results section started with the text on line 164 and with Figure 2. In my opinion, a short sentence like “The cell type, stain set, activator, activator concentration, and incubation time used in the screen were selected based on two pilot studies (ref supplemental for additional details)” could replace L102-163 and Figure 1. However, this is a matter of opinion and I ultimately leave the decision up to the authors.

Did you profile enough compounds in the pilot studies to definitively make the statements in L384-388? I would at least qualify this with something like “based on our pilot screens with X compounds”, or soften the language.

The discussion should include a section on limitations, and I recommend discussion of the limitations of a single replicate.

Open Access This Peer Review File is licensed under a Creative Commons Attribution 4.0 International License, which permits use, sharing, adaptation, distribution and reproduction in any medium or format, as long as you give appropriate

credit to the original author(s) and the source, provide a link to the Creative Commons license, and indicate if changes were made.
In cases where reviewers are anonymous, credit should be given to 'Anonymous Referee' and the source.
The images or other third party material in this Peer Review File are included in the article's Creative Commons license, unless indicated otherwise in a credit line to the material. If material is not included in the article's Creative Commons license and your intended use is not permitted by statutory regulation or exceeds the permitted use, you will need to obtain permission directly from the copyright holder.
To view a copy of this license, visit https://creativecommons.org/licenses/by/4.0/

Reviewer #1 (Remarks to the Author):

The manuscript "Cell painting in activated cells illuminates phenotypic dark space and uncovers novel drug mechanisms of action" by Zietek et al., performs the now well established Cell Painting assay to screen a collection of bioactive small molecule compounds in the A549 lung cancer cell line under standard cell culture conditions and also conditions which "activate" cells following phorbol myristate acetate (PMA) treatment. The article is well written and includes some nice methodology for quality control and assessment of a Cell Painting activity score. The major conclusions is that cell activation upon PMA illuminates new phenotypic "dark space" following compound treatment that would otherwise be missed under standard cell culture conditions.

General comments: While this is a well conducted study that make the important point that perturbation of cell culture systems influences how cells respond to drug treatment, and in turn will influence the ability of the Cell Painting assay to identify and cluster bioactive compounds by phenotypic profiling -this observation however is not unexpected.

We recognize that Reviewer 1 characterized the findings as "not unexpected," but respectfully note that demonstrating and quantifying an expected phenomenon at scale, with appropriate controls and validation, is itself a significant scientific contribution, particularly when it provides a framework the community can adopt and build upon. We also note that our analysis of target-class consolidation and deconsolidation under activation conditions reveals pathway-level insights that go beyond simple confirmation of a known effect.

We agree that it is not unexpected that a baseline drug treatment (i.e., PMA) will influence the ability to cluster bioactive compounds by phenotypic profiling; however, our study is the first to evaluate this effect in detail, quantify the results, and evaluate the effect in different cell lines and activators (both PMA and EGF). Therefore, we think the study as a whole merits publication in Nat Comm due to its overall breadth and depth of analysis.

Also, the magnitude to which PMA treatment reduces phenotypic dark space appears somewhat limited when using robust activity thresholds or 5% false discovery rate and while performance of drug mechanism-of-action clustering is improved upon PMA stimulation for some therapeutic target classes, it is reduced for other target classes. These results indicate that the approaches described and PMA stimulation specifically may only have a subtle effect upon illuminating phenotypic dark space and while this will likely uncover novel mechanisms-of-action other important mechanisms-of-action may be lost.

We think it is more important to consider the combined number of compounds that were phenotypically active under both PMA-stimulated *and* unstimulated conditions, and to compare that with the results from the unstimulated (classical CP) condition alone. At the 5% false discovery rate (FDR) threshold, combining profiles from resting and PMA-activated cells led to 1,343 active compounds at 1 µM, compared to 778 under the standard (resting-only) condition, a ~1.7-fold increase representing 565 compounds whose phenotypic activity was detectable only upon cell activation. This conservative estimate increases substantially at less stringent activity thresholds (Supp. Fig. S1B), confirming that the benefit of PMA activation is robust across the threshold range examined. The most meaningful comparison is between the combined (PMA + resting) dataset versus the standard (resting-only) condition, and the word "substantially" in our manuscript reflects this combined improvement, not the PMA-alone condition.

To make the abstract more accurate in this respect (using the more stringent 5% FDR rate to quantify phenotypes), we changed this sentence in the abstract:

Over 1,000 compounds exhibited phenotypes exclusively under PMA activation, establishing its advantage for MoA studies.

To the following:

The combined cellular CP fingerprints from PMA-activated and resting cells revealed phenotypes for 565 compounds that were inactive under the resting condition alone, establishing its advantage for MoA studies.

Specific points:
The authors state in the discussion that they "demonstrate that activating cells with PMA prior to CP analysis substantially expands the observable phenotypic space and enables the detection of otherwise inactive compounds", however the magnitude of phenotypic space expansion appears somewhat limited. As the authors present in Figure 1C : dark phenotypic space in combined standard and PMA (1micromolar) stimulated conditions is still extremely high at 72%, while with PMA treatment alone dark phenotypic space is 80% compared to 85% in standard cell culture conditions. These results would appear to contradict the authors claim that PMA activation "substantially" expands observable phenotypic space.

While the authors show in supplementary Figure 1B that observable phenotypic space can be significantly increased with setting a lower CP activity threshold, as the authors acknowledge, this comes at the cost of increased false positive activity and when using a 5% false discovery rate (supplementary figure 1B) the dark phenotypic space remains even higher at: 84% in combined standard and PMA (1micromolar) stimulated conditions; 93.2% in PMA treatment alone compared to 90.7% in standard cell culture conditions.

We agree that the dark space observed among the entire TargetMol library remained relatively high at 1 uM (at the 5% FDR rate, 7026 compounds were inactive in either condition, compared with 1343 active compounds in the combined PMA/noPMA condition, and 778 in the no-PMA condition alone). The number of active compounds increased by more than 2-fold at 10 uM (3018 inactive compounds, compared to 2075 active compounds in the combined PMA/noPMA condition, and 1357 in the no-PMA condition alone). This suggests that more than half of the phenotypic dark space observed at 1 uM was due to the relatively weak potency of most of these drugs in this library. In addition, many of the compounds have primary targets that are not present in mammalian cells, e.g., antibiotics, antivirals, etc. Many plant-derived natural products and compounds with with ambiguous mechanisms of action fall into this class. Thus, we argue that the important comparison is between the combined data (treated plus untreated) vs. standard (untreated) conditions alone; based on these numbers we contend that, while not unexpected, the ability of PMA activation to illuminate phenotypic dark space under the conditions described is indeed substantial.

Line 254: "The resulting phenotypic fingerprints consist of hundreds of cell features extracted from the segmented regions of the images for each cell and further processed using the HistDiff algorithm..". Can the author clarify here how many features were extracted per cell and whether

feature reduction methods has any impact on illuminating novel dark phenotypes in PMA activated cells? E.g. would more features reduce the dark phenotypic space?

We added a new section to the Methods that describes three different feature reduction methods that we applied and their impact on target classification. In terms of the question as to whether these feature reduction methods affected the illumination of dark space, we used the entire, non-reduced fingerprints to assess phenotypic activity as described in Figure 3.

Line 309: "Indeed, thanks to PMA efficacy, we can clearly observe the phenotypic effect of PKC, MEK, and ERK regulators in PMA-treated cells, among others, giving us confidence in the screen results (Fig 4D)". I agree the phenotypic effect of these compounds are enhanced in PMA-treated cells which are expected and encouraging with regards to the screening set up. Are the activity of other unexpected target classes enhanced or diminished in PMA stimulated conditions which may provide some novel insights into particular compound classes?

In addition to the kinase pathway mentioned above, we also point out that the glucocorticoid modulator class becomes significantly more consolidated with PMA activation. We discuss this briefly in the results section with the following statement and associated references:

GR-targeting compounds, which displayed more heterogeneous phenotypes in resting cells, clustered tightly in the presence of PMA, consistent with literature describing antagonism between GR signaling and PKC-induced transcription factors such as AP-1 and NF-kappaB [21,22]

Line 378 "In contrast, other target classes became less consolidated under PMA treatment (Fig.4F, red), reflecting greater phenotypic diversity upon PKC activation." Could this phenotypic diversity reflect a lack of activity/potency resulting from loss of the primary mechanisms-of-action of compounds that are present in the standard culture conditions, thus now amplifying more subtle off-target compound activities?

We are grateful for the reviewer for making this point and have added this point at the relevant section as shown below:

Furthermore, the decrease in consolidation may reflect a lack of activity resulting from loss of the primary mechanisms-of-action of compounds that are present in the standard culture conditions, thus now amplifying more subtle off-target compound activities. Together, these findings suggest that acute pathway activation reorganizes the phenotypic landscape, consolidating the phenotypes of compounds targeting convergent pathways, while dispersing the phenotypes of compounds whose activity becomes context-dependent under activation. This highlights the value of multi-point perturbation—such as combining functional compounds with activators—in revealing hidden structures in phenotypic response space and mapping conditional dependencies between signaling pathways.

If this is the case this could confound the utility of some cell activating conditions to effectively identify and predict important drug mechanisms of action from Cell Painting profiles.

While it is true that "deconsolidation" of target classes upon activation would decrease the ability to identify drug mechanisms if the fingerprints under the activated condition were considered

alone; however, our work suggests that the combined fingerprints from the activated and resting conditions lead to a net improvement in the ability to predict mechanisms of action. We also think that our methodology and the results themselves are interesting and worth reporting, and feel that the use of entropy analysis to investigate target class consolidation under the two conditions across the entire library led to insights into pathway-level connections that we hope will inspire future systems-level approaches to the analysis of cell painting data obtained under other specific perturbations.

Reviewer #2 (Remarks to the Author):

The hypothesis that certain phenotypic responses to compound exposure are only observable under particular cell states is interesting, and worth investigating. However, I have concerns about the overall experimental design, particularly the apparent lack of replicates, and more information on the experimental design is needed to properly evaluate whether the conclusions derived from analysis are sound. The paper structure could also be re-worked to improve readability by focusing more on the main results, and moving details on the pilot / qa/qc to the methods and supplementary.

Large comments:
- Concerned about the lack of replicates. There are not enough experimental design details provided to evaluate how big of an issue this is. There are substantial plate and well position effects in Cell Painting screening studies. Without replicates that are properly randomized across well positions and plates, it is difficult to determine whether an effect is statistically significant and whether it is a true biological effect or confounded by technical factors. Were activated and non-activated samples on different plates? Were the well positions of the same compound the same under activation versus non-activation conditions? Since well position and plate effects are ubiquitous, knowing these details are important for evaluating the downstream data analysis.

We agree on the importance of stating explicitly the format of our controls and appreciate the reviewer's attention to this concern. The following was added to the beginning of the results section describing the large-scale screen.

Each plate contained 38 vehicle-only control wells (including a combination of DMSO-only and PMA-only wells in both the PMA-treated and untreated plates) in randomized positions (Figure 2A), providing extensive within-plate references for normalization and quality assessment. All controls were distributed within each plate using a mixed integer linear program (MILP) designed specifically to minimize positional and batch effects. The noPMA and PMA conditions were processed in parallel batches with identical plate maps, ensuring that any given compound occupied the same position in the two conditions, thereby controlling for systematic positional effects between conditions. We implemented additional quality control methods to confirm activation and verify reproducibility among the positive controls, as described in the Supplemental Methods.

- 3 of 5 figures describe the experiment (pilot, overview, qa/qc) while only two focus on the main result of the paper. The amount of text is also proportional here. Suggest greatly condensing the first 60% of the paper (ie. one figure, proportional amount of text), enabled by ample use of the supplementary materials, to put more focus on the main results.

We thank the reviewer for this suggestion and agree that the initial proof-of-concept studies outweigh the main part of the paper. We think the pilot study in which we select the activator (PMA vs. EGF), cell line, timing, and activator concentration is important to include in the main body in that it lays the groundwork for the main screen. However, to streamline the manuscript as per this suggestion, we have moved the qa/qc section (originally Fig. 3 and the associated discussion) to the Supplemental Information.

- Many methodological details are repeated across the methods and results section. The paper would be more readable if this was reduced, for example L353-359 can be included in only the methods and replaced with a short statement describing what the main finding of Figure 5A is and why it matters, rather than how it was produced.

We have removed redundant descriptions of methods, especially those contained in the figure captions, which has streamlined the Results section considerably.

Small comments:
- Figure legends are much too long and contain a mix of methods, results, and interpretation

We agree with this critique and have significantly edited the figure legends to remove redundant descriptions of experimental methods. We have also split up the original Figure 4 into two separate figures (now Figures 3 and 4), which we also think will clarify this part of the Results section.

- L354-359: Don't understand the dimension reduction to UMAP components prior to similarity matrix calculation. UMAP has issues and should be used with many grains of salt; why not use a more reliable method like PCA or just use raw features if the next step is to compute profile:profile similarity and not to directly visualize in low dimensions?

We acknowledge that UMAP has some limitations, particularly its sensitivity to parameterization and its primary use as a visualization tool. For this reason, we explicitly benchmarked UMAP-based composite features against PCA and non-composite feature-reduction strategies in a side-by-side, quantitative manner using downstream KS-test performance rather than visual separation alone. Across multiple conditions and projection depths, UMAP (at ~100 components) consistently preserved or improved target-class discrimination relative to PCA and raw or minimally reduced feature spaces, while substantially reducing dimensionality and computational burden. In this context, UMAP functions analogously to PCA as a composite feature generator, but is better suited to our data due to its ability to model nonlinear structure and mitigate collinearity inherent in high-dimensional Cell Painting profiles. We also note that we generated the UMAP projections using a fixed random seed to address the stochasticity issues associated often associated with UMAP.

To clarify this for the reader, we have added the following to the Feature Reduction section in Methods.

In order to streamline downstream data analysis and reduce the dimensionality of the feature set, we investigated three approaches using the K-S test to assess each feature set's ability to preserve or improve target-class discrimination. The initial HistDiff-processed phenotypic fingerprints consisted of 5,880-feature vectors. The two (1 µM and 10 µM) sets of the original

5,880-feature HistDiff fingerprints for each treatment condition (compound with or without PMA) were included such that each compound dose treatment had two representatives. To consider the combined condition for each compound dose, the two (with or without PMA) 5,880-feature fingerprints were concatenated, resulting in an 11,670-feature fingerprint. Uninformative features were then removed as described in [19], starting with removal of features with zero standard deviation across all treatments. Then, using the 'remove multicollinearity' function from the Pycaret package (version 3.3), all feature pairs with Pearson correlation coefficients greater than a threshold parameter of 0.8 were flagged, thereby reducing redundancy between the features. Each pair member with the highest mean correlation to all other features was removed, resulting in the 482-feature CP fingerprint for each condition. When looking at the combined view of both conditions (with and without PMA), each set of 482-feature vectors for each compound dose was concatenated, resulting in a 964-feature CP fingerprint for each compound dose in the study. These 964-feature CP fingerprints derived from this collinearity reduction approach were used for clustered heatmaps and for quantifying the strength of phenotypic response in the study.

For computationally intensive analyses such as clustering of the entire dataset, we set out to evaluate further data reduction methods, specifically principal component analysis (PCA) and UMAP[20]. For each feature reduction method (colinearity reduction, PCA, and UMAP), we used the K-S test to compare the pairwise similarities of the compound fingerprints within each annotated target class with their similarities to all other compounds outside that class. For each data reduction method, we counted the number of target classes whose significance was at or less than a *p*-value of 0.01. UMAP composite features were generated using the correlation metric and neighbors parameter of 10, with all other parameters left to default including a setting the initial seed to a fixed integer of 15. The resulting 100 UMAP composite features produced 222 significantly enriched target classes, which performed as well as the 964-feature fingerprints obtained from the collinearity method described above (219 enriched classes) and much better than PCA (197 enriched classes), even at a threshold of 99% of the variance captured (this threshold for the PCA required 292 composite features). We therefore used the 100 UMAP composite features to perform HDBSCAN clustering [33] and other computationally intensive analyses.

- The "JUMP stain set" is the Cell Painting assay, which refers to the particular set of stains outlined in Bray et al and Cimini et al (both Nat Prot). Suggest calling it the Cell Painting stains to avoid confusion (examples: L120, L129, L137)

We thank the reviewer for this observation and have changed all references in the main text from "JUMP stain set" to "Cell Painting stain set", and added the following sentence to clarify:

The third set in the pilot and the screen were stained as described by the JUMP Consortium[14] using their Cell Painting stains (designated the Cell Painting or JUMP stain set) and protocol version 3 (with one assay modification: the drug incubation time was reduced from 48 h to 24 h to limit drug secondary effects).

(Reference 14 cites Bray and Cimini Nat Prot).

REVIEWERS' COMMENTS

Reviewer #1 (Remarks to the Author):

I thank the authors for their responses and clarifications to the original reviewers comments and the efforts they made to revising the manuscript. I restrict my comments and response to the following major outstanding points.

In response to the original comments that the results are not unexpected the authors responded: "We agree that it is not unexpected that a baseline drug treatment (i.e., PMA) will influence the ability to cluster bioactive compounds by phenotypic profiling; however, our study is the first to evaluate this effect in detail, quantify the results, and evaluate the effect in different cell lines and activators (both PMA and EGF)". I do not disagree with the authors that they are the first to evaluate the effect in detail, however I remain of the opinion that this work does not represent a significant enough novel conceptual advance to merit publication in Nature Communications.

In response to the original comment: The magnitude to which PMA treatment reduces phenotypic dark space appears somewhat limited.... The author responded:
"We think it is more important to consider the combined number of compounds that were phenotypically active under both PMA-stimulated and unstimulated conditions, and to compare that with the results from the unstimulated (classical CP) condition alone. At the 5% false discovery rate (FDR) threshold, combining profiles from resting and PMA-activated cells led to 1,343 active compounds at 1 μM, compared to 778 under the standard (resting-only) condition, a ~1.7-fold increase representing 565 compounds whose phenotypic activity was detectable only upon cell activation. This conservative estimate increases substantially at less stringent activity thresholds". While the 1.7 fold increase in phenotypic activity of compounds detected upon cell activation is encouraging total dark phenotypic space in combined standard and PMA stimulated conditions is still 72% compared with unstimulated dark phenotypic space at 85%. Reducing stringency threshold and increasing concentration of drugs to 10uM will certainly result in less phenotypic dark space but at the cost of high error rates, multiple off-target activities at non physiologically relevant concentrations. Thus the results while positive still seem somewhat incremental in reducing phenotypic dark space particularly if the methods would be applied in practice at earlier stages of drug discovery where compound potency is typically low.

When it comes to evaluating the overall significance of our results, the discrepancy seems to be a matter of perspective. While the reviewer is focusing on the improvement from 85% dark phenotypic space in unstimulated (traditional) cells to the 72% we obtain with our method, we are considering the fold increase (1.7-fold) between the two methods and the actual number of new compounds that were dark and become illuminated (565). We contend that the large number of phenotypically dark compounds in our known bioactives library has more to do with the fact that most of the compounds in this library are not potent enough to engage their targets at 1 uM, or simply have targets that are not present in these cells.

I agree that the methodology and the results themselves are interesting and worth reporting but i think this work would be better suited to a specialized chemical biology or drug screening journal.

Reviewer #2 (Remarks to the Author):

The paper is much improved and I appreciate the additional details about plate and well position layout - I am reassured that the experimental design does not confound compound effects with these technical factors. I am still skeptical about the single replicate for each condition, but appreciate that the large number of profiled compounds still enables detection of large-scale patterns.

We acknowledge this limitation and have added the following sentence in bold below to the manuscript.

As this chemical library was tested in two drug concentrations (1 and 10 µM) in resting and PMA-activated cells, and several plates were tested in replicas for quality control (QC) reasons, the combined number of conditions tested sum up to almost 50,000 individual wells. ***At this scale, obtaining technical replicates of each compound was cost-prohibitive, prompting the development of a rigorous quality control pipeline to ensure reproducibility.***

I have a few additional comments:

Cell Painting should be capitalised in the title and key words
Done.

I appreciate that the authors moved much of the QA/QC discussion to the supplementary materials, however I think that the paper would be even more compelling and readable if the results section started with the text on line 164 and with Figure 2. In my opinion, a short sentence like "The cell type, stain set, activator, activator concentration, and incubation time used in the screen were selected based on two pilot studies (ref supplemental for additional details)" could replace L102-163 and Figure 1. However, this is a matter of opinion and I ultimately leave the decision up to the authors.

We thank the reviewer for these comments and agree that, in principle, these results could be moved to the SI without detracting too much from the narrative. However, we think the results of this pilot study lay important groundwork for the rest of the manuscript in that they show how we establish the best activation conditions for the large-scale screen.

Did you profile enough compounds in the pilot studies to definitively make the statements in L384-388? I would at least qualify this with something like "based on our pilot screens with X compounds", or soften the language.

We have changed that section as recommended by the reviewer to the following:

Based on our pilot studies with 72 compounds representing 18 different mechanistic classes, a systematic comparison of different activators, concentrations, and timing revealed that 4 h treatment with 50 nM PMA provided optimal cellular responses while maintaining cell viability.

The discussion should include a section on limitations, and I recommend discussion of the limitations of a single replicate.

We agree that we should address the lack of technical replicates and have added the following to the Discussion Section:

Cell Painting data are inherently noisy, arising from biological variability at the single-cell level, staining heterogeneity, and positional effects within and across plates. Technical replicates are therefore valuable

for distinguishing true compound-induced phenotypes from stochastic variation. A key limitation of this study is that individual compound treatments were not replicated. Screening ~8,500 compounds at two concentrations in both resting and PMA-activated states already required nearly 50,000 wells; incorporating even duplicate measurements would have doubled the screen size, making it cost-prohibitive. To mitigate this, we employed 38 randomized vehicle-only control wells per plate, a MILP-optimized plate layout to minimize positional and batch effects, identical plate maps across conditions, and a multi-checkpoint quality control pipeline including a machine learning-based activation classifier and the QCGauntlet visualization tool. While these measures provide confidence in the reproducibility of our data, the absence of technical replicates means that the activity of any individual compound should be validated independently before drawing mechanistic conclusions, as we demonstrated for 2-methoxycinnamaldehyde and the GR cluster
